# Moral licensing, instrumental apology and insincerity aversion: Taking Immanuel Kant to the lab

**DOI:** 10.1371/journal.pone.0206878

**Published:** 2018-11-08

**Authors:** Elias L. Khalil, Nick Feltovich

**Affiliations:** Department of Economics, Monash Business School, Monash University, Clayton, Victoria, Australia; Universidad Loyola Andalucia, SPAIN

## Abstract

Moral licensing, equivalently called “self-licensing”, is the instrumental use of a Good Act to cover up a Bad Act. This paper’s thesis is that “instrumental apology” i.e., bad-faith apology, is a case of moral licensing. A decision maker may issue an apology (Good Act) after committing a Bad Act, but if the decision maker uses the apology instrumentally, he or she is using the apology to justify the Bad Act. Hence, the apology is insincere. Sincerity is the fine line between a good-faith apology or, more generally, a Good Act, on one hand, and an instrumental apology or, more generally, moral licensing, on the other. In this light, moral licensing should be separated from genuine apology that attains moral equilibrium, which is called in the literature moral “self-regulation’ and “conscience accounting.” According to Kantian ethics, not just the consequences of an act matter, but also the sincerity with which the act was conducted. This pits Kant against the utilitarian view, which downplays intentions and focuses on consequences. We take Kant to the lab. Participants play a modified ultimatum game, where proposers in some treatments have the option of issuing apology messages and responders have both costly and costless options for rewarding or punishing proposers. We introduce different treatments of the apology message to allow responders to form doubts about the sincerity of the apology messages. Our results support the Kantian position: responders, once they become suspicious of the sincerity of the proposers’ apology, exhibit “insincerity aversion” and punish proposers.

## Introduction

In *The Missionary Position*: *Mother Teresa in Theory and Practice*, Christopher Hitchens [1] criticizes Mother Teresa for accepting gifts from disgraced bankers, such as Charles Keating, and ruthless dictators, such as the ex-ruler of Haiti, Jean-Claude Duvalier. To Hitchens, Mother Theresa sold “indulgences” to unsavory characters. Critics faulted indulgences in the Middle Ages on the ground that many people tended to use them as instrumental apologies, i.e., inauthentic apologies to receive benefits from God.

Mother Teresa defended herself: she did not sell indulgences. Rather, the donors regarded their charitable donations as expressions of sincere remorse for their bad deeds [2], and it would be churlish to reject gestures of sincere apology, no matter their source. For Mother Teresa, the villains, in making the donations, were regaining what is here called “moral equilibrium”, which is *somewhat* equivalent to what Sachdeva, Iliev, and Medin [3] dub “moral self-regulation”, Brañas-Garza, Bucheli, Espinosa, and García [4] call “moral cleansing”, and Gneezy, Imas, and Madarász [5] name “conscience accounting”. Hitchens retorted: Keating’s and Duvalier’s apologies were not genuine apologies to restore their moral equilibrium; rather, they were “moral licenses”—instrumental apologies—Good Acts abused to camouflage or eschew responsibility for Bad Acts.

From this exchange, it is apparent that Mother Teresa and Hitchens agree about the principle under focus: the insincere use of a Good Act such an apology amounts to an “instrumental apology,” defined as a bad-faith apology. A review of the literature shows that apologies backfire if recipients see them cynically, i.e., as instrumental apologies motivated to advance the self-interest of the utterer. So, while Hitchens and Mother Theresa disagree on the specific cases of Keating and Duvalier, they concur that sincerity delineates genuine apologies from instrumental ones. This paper goes one step further: it classifies moral licensing within the camp of instrumental apologies and, symmetrically, classifies moral equilibrium within the camp of sincere apologies.

The task of this paper is twofold. The first task, undertaken in the first section, is to lay out a conceptual framework. The second task, undertaken in the rest of the paper, is to present the results of an experiment documenting moral licensing—in particular, how people are ready to punish others suspected of a particular form of moral licensing: an instrumental apology.

## The conceptual framework

### The thesis

This paper advances the thesis that the phenomenon called “moral licensing” [6] is fundamentally the same as the instrumental apology. Both are based on insincerity. That is, sincerity is the wedge that separates an instrumental apology or, more generally, moral licensing, from a genuine apology or, more generally, moral equilibrium. While a genuine apology setiment restores moral equilibrium, an instrumental apology sentiment is a form of moral licensing. Hence, a moral licensing sentiment cannot restore moral equilibrium—and in fact pushes the sentiment further away from moral equilibrium. Currently, as shown below, much of the literature conflates moral licensing and moral equilibrium [3]. In particular, the literature fails to ground instrumental apology in moral licensing, and hence fails to distance moral licensing from moral equilibrium. While moral equilibrium is restored by, among other things, sincere apology, instrumental apology and moral licensing belong to another camp, namely, gestures and acts that are insincere. The extrication of instrumental apology, and generally moral licensing, from the concept of moral equilibrium is the key conceptual innovation of this paper.

Sincerity, as both Mother Teresa and Christopher Hitchens concur, is the central feature of genuine apology that restores moral equilibrium. In this regard, both are in accord with the spirit of Immanuel Kant’s ethical theory: sincerity matters and, hence, for an apology to be accepted it must be authentic rather than instrumental [7, 8]. Converesly, for utilitarians and their fellow travelers such as the welfarist economists, sincerity does not matter or, if it does, it matters only as a preference like any other. For utilitarians, what ultimately matters is the consequence of the action rather than the intention behind it.

The debate between Kantian theorists and utilitarian economists is not an esoteric debate. It animates much of political discourses and public debates about apologies and moral licensing, from Nisan and Horenczyk [9] and Monin and Miller [10], to the more recent social psychology and business ethics literature [3, 6, 11–13]. Management theorists recently started to pay attention to moral licensing to analyze counterproductive work behavior [14], abusive supervisors who are otherwise high-performers [15], and low provision of family support [16]. Economists should also pay attention to moral licensing as insincerity; insincerity may explain why buyers walk away from sellers who resort to practices such as “hard bargaining” and “price gouging” [17, 18].

### Moral licensing and related concepts

What is moral licensing? Khan and Dhar provide a definition of the phenomenon, which they call the “licensing effect”:

[A] prior virtuous decision boosts the relevant self-concept, which mediates the preference for a luxury option. … [C]onsumers may be unaware of how their prior decisions influence their subsequent choices. In other words, the process underlying the licensing effect may be largely nonconscious. (see [11], p. 259)

This conforms with how Merritt, Effron, and Monin [6] define one mechanism of moral licensing:

[M]oral self-licensing … occurs when past moral behavior makes people more likely to do potentially immoral things without worrying about feeling or appearing immoral. (see [6], p. 344)

This mechanism, which Merritt, Effron, and Monin [6] call the “moral credit” model, differs from another mechanism, which they call the “credentials” model [4]. For the “moral credit” model to work, the intention of the Bad Act must be unambiguous for the receivers and the actor, but the actor appeals to another Good Act as a stock of moral capital to provide a license for performing the Bad Act. For the “credentials” model to work, the intention of the Bad Act must be ambiguous for the receivers and usually also for the actor. This ambiguity opens a back window for the actor: the actor appeals to past Good Acts as credentials to deceive the receivers and usually also the self into thinking that the intention behind the Bad Act is also good.

The difference between the two models is rather minimal: while the actor in the “moral credit” model is fully aware that the current act is a Bad Act, the actor in the credential model might be a victim of self-deception [19, 20]. But where there is no self-deception, the difference between the two models collapses.

The distinction between the two models of moral licensing highlight two caveats. First, self-deception or, in particular, a lack of self-awareness is not a necessary condition of moral licensing. Moral licensing takes place as long as one—whether consciously or nonconsciously—uses a Good Act to justify or explain away a Bad Act. Hence, contrary to the assumption common in the literature [13], the distinguishing feature of moral licensing is not that the act is bad at the nonconscious level.

To note a parallel insight, the issue of consciousness or awareness is as irrelevant to moral licensing as it is to rational choice: that is, rational choice (and the moral licensing choice) takes place irrespective of whether the actor is conscious of the choice.

The second caveat, the definitions in the literature at large [21] use terms—e.g., “immoral things”, “virtuous decisions”, and non-virtuous acts that involve a “luxury option”—without the necessary hard work of defining the terms “morality,” “virtue,” and why a “luxury option” is immoral. The literature provides little precision of what these terms mean and, at best, provide only vague theoretical underpinnings. This imprecision and vagueness have given rise to two *confusions*:

1. When one refrains from self-indulgence in one act, one normally compensates (what the economists regard as substitution) with indulgence in another act. The literature seems to confuse the legitimate act of substitution with the illegitimate act of moral licensing.
2. It is often the case that when one commits an immoral act, one tries to redeem oneself with an apology, which may even involve gifts, in order to restore “moral equilibrium”. When the receivers of the gifts, given they are truthful, perceive the apology as genuine, they usually accept it and allow the one to restore one’s moral equilibrium. The literature seems to confuse the praiseworthy seeking of moral equilibrium with the deplorable act of moral licensing.

To avoid these two confusions, we propose a stricter definition of moral licensing than that found in the literature. Namely, we define moral licensing as primarily based on *insincerity*. This definition allows us, first, to distance moral licensing from normal substitution and, second, to distance moral licensing from seeking moral equilibrium.

### Upon what does moral licensing rest?

Conversely, regarding the timing of the act and the content of act, our proposition about the centrality of sincerity affords a broader definition of moral licensing than that common in the literature. On our definition, the timing of the Good Act and Bad Act can be reversed. That is, the issue of temporal order is not central to the definition of moral licensing. Further, on our definition, the content of the Good Act and the Bad Act need not be pecuniary, substantive deed—it could instead be words of kindness ase well ass words of unkindess. That is, the issue of pecuniary benefit is not central to the definition of moral licensing.

Stated differently, the conceptual innovation of this paper rests on the proposition that the core of moral licensing neither depends on the timing of Good and Bad Acts, nor on the pecuniary, substantive content of the Good Act. Instead, moral licensing ultimately rests on sincerity or authenticity. An act is moral licensing where the actor uses the Good Act insincerely, i.e., uses it instrumentally to attain a purpose other than the expressed purpose.

To justify the centrality of sincerity, we can distil the structure of moral licensing (Hitchens’s criticism), where arrows denote the temporal sequence of events rather than causality:

Statement 1a: defining moral licensing (Hitchens’s criticism):

Bad Act at t_1_ → Good Act at t_2_ → Agent misuses the Good Act at t_2_ to cover up the Bad Act at t_1_ or to put the Bad Act at t_1_ in a better light.

Statement 1b: defining moral licensing (Hitchens’s criticism):

Good Act at t_1_→Bad Act at t_2_→Agent misuses the Good Act at t_1_ to cover up the Bad Act at t_2_.

Now, let us distil the structure of moral equilibrium (Mother Teresa’s defense):

Statement 2a: defining moral equilibrium (Mother Teresa’s defense):

Bad Act at t_1_ → Good Act at t_2_ → Agent uses the Good Act at t_2_ to express sincere remorse or ask for genuine forgiveness concerning the Bad Act at t_1_.

Statement 2b: defining moral equilibrium (Mother Teresa’s defense):

Good Act at t_1_→Bad Act at t_2_→ Good Act at t_3_ → Agent uses the Good Act at t_3_ (and never the Good Act at t_1_) to express sincere remorse or ask for genunine forgiveness concerning the Bad Act at t_2_.

To clarify Statement 2a, it rules out the case when the decision maker undertakes a Bad Act because he or she intends to do a Good Act in the future. Such a Good Act would be a moral license and, hence, contrary to Statement 2a.

We can draw general observations concerning the above set of statements:

1. While the time sequence of the Good Act and the Bad Act is irrelevant for moral licensing, the Good Act must follow the Bad Act for moral equilibrium—i.e., to restore one’s moral standing through genuine remorse or apology. This characterization of moral equilibrium is consistent with how Brañas-Garza, Bucheli, Espinosa, and García [4] define moral equilibrium, which they call “moral cleansing”: “immoral behaviour has a negative effect on moral self-worth. After engaging in bad deeds, people follow a moral behaviour to recover the lost self-worth; this mechanism is the so-called moral cleansing behaviour”—which is at the origin of physical cleansing in many religious rituals [22]. This characterization of moral equilibrium is also consistent with how Gneezy, Imas, and Madarász (see [5], p. 2645) define moral equilibrium which they call “conscience accounting”: “people donate to charity … to offset a feeling of guilt associated with recent bad actions”;
2. This paper, as mentioned at the outset, differs from the literature in one regard. Namely, the literature generally treats moral cleansing and moral licensing as two *facets* of the same supposed moral equilibrium [3–5]: As for the moral cleansing facet, one usually undertakes a Good Act to cleanse the conscience. As for the moral licensing facet, one uses instrumentally a Good Act to justify a Bad Act. However, these supposed facets of moral equilibrium are asymmetrical. While the public and spectators welcome genuine apologies as expressed in moral cleansing (moral equilibrium), they deplore and condemn instrumental apologies as expressed in moral licensing as this paper’s experiment attempts to confirm. If one facet (the moral cleansing of conscience that restores moral equilibrium) were symmetrical with the other facet (moral licensing), we would face the anomaly of how to explain the asymmetrical reaction of spectators.
3. The Good Act can take different forms: for example, it could be a charitable deed, but it could also be a verbal apology/appreciation.
4. The core criterion for delineating moral licensing from moral equilibrium relates to the use or misuse of the Good Act. That is, keeping with the conceptual innovation, neither the timing of the act nor its particular cotent is essential to the definition of moral licensing.

In the cases of Keating and Duvalier, Hitchens is leveling Statement 1a, while Mother Teresa is asserting Statement 2a. Nonetheless, both implicitly subscribe to the proposed definition that sums up the above set of statements: A Good Act stops being about moral equilibrium and becomes moral licensing once the agent uses it instrumentally/insincerely, i.e., for a purpose other than the act’s stated or implied purpose.

Brañas-Garza, Bucheli, Espinosa, and García [4] and Gneezy, Imas, and Madarász [5] conduct experiments that document moral equilibrium. While each study is based on different experimental designs, both studeis show that participants tend to commit good deeds after failing to commit pro-social choices (Statement 2a). In contrast, this study separates the phenomenon of moral licensing from moral equilibrium and asks: What happens when the Good Act is no longer about moral equilibrium but is instead used instrumentally, i.e., when does the “Good Act” become moral licensing?

### Sincerity and insincerity

The determination of whether a particular act is moral licensing, as opposed to moral equilibrium, is difficult to make in practice because one cannot observe insincerity. It is not straightforward for an observer to determine whether an act is sincere: Is a person misusing a Good Act—i.e., using it instrumentally for a purpose other than its stated or implied purpose? Or is the person properly using a Good Act—i.e., using it as a sincere gesture? Before we can examine this question, we first need to define sincerity:

Sincerity: The action of an actor is sincere when the actor’s stated or implied intention faithfully matches his or her hidden, true intentions (see [23], pp. 114–117).

Hence we define insincerity as a “mismatch” between stated intention, on one hand, and true intention, on the other: one is insincere when one does not state one’s full set of true intentions. For example, someone donating money to charity because of both altruistic and reputational concerns would be insincere if she claimed it was done exclusively from altruism. In Hitchens’s view, Keating and Duvalier were insincere because their Good Acts were declared to be driven exclusively by altruism, when they were driven, at least in part, by a desire to rescue their reputations as seen by public opinion.

There are two caveats about the definition of insincerity as a mismatch between stated and true intentions. First, one may feel regret for previous misdeeds and express remorse by giving to charity even if the “stated” purpose of the charity may have nothing to do with one's previous Bad Acts. In such cases, there is a difference in purposes or consequences of the acts, but there is no mismatch in the sense that matters for our definition. In the sense used here, the mismatch must be regarding intentions, not motivations. Second, an act can be sincere without being a Good Act. For example, in acts of vengeance driven by dark emotions such as hate and schadenfreude, the actor can be sincere about their intention. Thus the sincerity/insincerity dichotomy is orthogonal to the Good-Act/Bad-Act dichotomy.

We propose, in short, that insincerity-as-mismatch is the core defining feature of moral licensing and, hence, what delineates moral licensing from moral equilibrium. Moral equilibrium is about the sincere attempt to atone for Bad Acts by the commission of Good Acts, whereas moral licensing is the insincere use of Good Acts to help make excuses for past Bad Acts or to enable undertaking of new ones. Insofar as people resent and deplore moral licensing, we argue that the resentment and censure arise because of “insincerity aversion.”

This proposed framing of the question does not agree with Xiao (see [24], pp. 169–171). She maintains that an apology can combine the instrumental (insincere) motive with what she calls the “spontaneous” motive, i.e., sincere desire to express remorse. However, spectators usually tend to categorize acts dichotomously: any act must be either sincere or insincere. This tendency is probably the outcome of the fact that spectators need to make a binary decision: they must either judge an act as aimed at moral equilibrium or reject it as moral licensing.

### Review of the literature

The literature on moral licensing does not usually ground it in insincerity aversion. Consequently, the literature fails to see that moral licensing and ill-received apologies are two instances of the same phenomenon. As we argue here, insincerity aversion is the common thread between the two. Just as insincerity causes apologies to backfire, insincerity also explains why people find moral licensing reprehensible. A Good Act—such as giving a gift or donating to charity—would backfire if used insincerely, as in the case of instrumental apologies. Indeed, Mother Teresa claimed that the donations to her charity by notorious figures were apologies—by which she meant that the donations were well-intentioned apologies. Likewise, Hitchens’ critique of Mother Teresa stands if he can show that the donations were insincere apologies, i.e., used as moral licenses to obfuscate bad deeds. His critique falls otherwise.

The insincerity aversion phenomenon involves some overlaps with “lying aversion” and “guilt aversion” [5, 25, 26]. If the act of lying makes the deciever burdened with the sentiment of guilt, the deceiver feels insincere. But the sheer act of “lying” does not connote the key variable that gives rise to guilt and, similarity, to insincerity. The act of lying need not give rise to guilt since it need not involve insincerity. For example, one may lie about *facts* such as whether one has earned a bonus, visited an ailing grandmother, or attended the funeral of a friend. Without any of these lies involving the intention of gaining a moral standing, enhance moral capital, or attaint public applause, they do not involve insinerity; they might be simply “white lies” in the sense of innocuous lies that helpe the person avoid awkward social moments. But once such lies are told to gain moral capital, the lies are insincere and, hence, can be classified as moral licensing. What matters for insincerity aversion, and for the rise of guilt, is lying about intentions in the sense of making excuses for Bad Acts.

The proposed definition of moral licensing as insincerity in the sense of covering up bad intentions allows us to account for diverse phenomena that the literature has not traditionally considered part of moral licensing. Conversely, the definition allows us to exclude a whole class of phenomena that are often mis-identified as moral licensing. The definition allows us, first, to exclude dishonest or unethical behavior *per se* [27, 28]. Insofar as such behaviour does not involve the covering up of one’s intentions, it is not moral licensing. Second, the definition allows us to exclude the open defense of dishonest acts on the basis of helping the poor, as in the case of Robin Hood [29, 30]. The defense of such dishonest acts is usually “justified” on the on the basis of restoring equity [31]. Similarly, the telling of “white lies” can be excluded as such lies are told to benefit the hearer rather than the teller [32, 33]. Further, the grounding of dishonest acts in some notion of social justice usually involves a hard choice: a choice between two compelling principles. Such a choice differs from moral licensing, which involves the covering up of one’s true intentions.

There are also acts, excluded by our definition, that might appear to be examples of either moral licensing or moral equilibrium but are neither. They can be explained without any need to invoke sincerity. To illustrate, it is sufficient to discuss two studies. The first study by Sachdeva, Iliev, and Medin who write:

Researchers looking at expenditure surveys have noted that when charitable giving becomes financially more valuable, because of changes in the tax code, churchgoers tend to give more and also attend church less …. Others have found that when advisors disclose a conflict of interest to their clients, they feel licensed to give even more biased advice. (see [3], p. 527)

The case of disclosure of conflict of interest is arguably one of moral licensing, as the Good Act of disclosing a conflict is used to justify the subsequent Bad Act of giving biased advice. However, it is far from clear that the case of churchgoing behavior involves either moral licensing or moral equilibrium. The finding that a change in the tax code leads to an increase in monetary donations alongside a decrease in church attendance can be explained by the usual substitution effect, i.e., how rational actos substitute between goods that arises from a change in relative prices. The increase in donations need not be used as ex post rationalization. A churchgoer can clearly declare that he is expressing his or her devotion differently, depending on the relative costs of different expressions.

The second study, by Effron, Jessica, Cameron, and Monin [12], analyzes political incorrectness through the lens of moral licensing. They show that endorsement of US President Barack Obama “licenses” one to favor whites [6]. This study follows an earlier one by Monin and Miller [10] which found that merely choosing an African-American—who was the most qualified applicant—for a hypothetical job increases the likelihood that participants would describe a subsequent job as better suited for whites. In the study by Effron, Jessica, Cameron, and Monin [12], though, it is not clear that moral licensing is occurring. It could be the case that many whites, when they endorse Obama, i.e., have the opportunity to take a non-prejudiced position, later feel free to express their bold opinions on racial questions, unafraid of being labeled “politically incorrect”.

Put differently, if someone is actually racist, but makes some anti-racist statements to justify other racist behavior, this behavior is appropriately labelled as moral licensing. But in the study by Effron, Jessica, Cameron, and Monin [12], someone could be fundamentally non-racist, but make some (extra) anti-racist statements to avoid being falsely accused in future. The latter case is not moral licensing, but rather an attempt to signal the quality of one’s opinion. Insofar as signaling does not invoke sincerity orcerity, it falls outside the focus of moral licensing.

To our knowledge, this paper is the first to ground moral licensing squarely in insincerity aversion. There are very few experiments or studies on the link between moral licensing and insincerity. There are, however, many experiments and studies that ground the ineffectiveness of apologies in insincerity. In the psychological literature, psychologists have studied the consequences of insincere apologies as opposed to sincere ones [34, 35], and have also recorded that insincere gestures usually engender negative outcomes [36–39].

In the economics literature, Fischbacher and Utikal [40] find that when agents harm others and issue an apology to them, the harmed others lower their punishment only if the intention behind the harm is ambiguous. When the harm is unambiguously intended, apologies backfire: the harmed others fully punish the perpetrators. Similarly, in the management literature, Brinke and Adams [41] find that customers care about the sincerity of firms issuing apologies. They analyze 29 corporate apologies to examine their effect on market returns. Using the help of two trained coders of nonverbal and verbal elements using a method based on the Facial Action Coding System developed by Paul Ekman and his collaborators, Brinke and Adams classified the facial affective expressions of company representatives as they apologized. They found that if the facial expression showed deviant or inappropriate emotions, such as a smiling face, market returns for the company were negative for many months after the apology. However, if the facial expressions were perceived as displaying sincere remorse, market returns for the company instead rose for many months after the apology.

Two other studies show how apologies may backfire, but ask different questions. In the first, Van Dijke and De Cremer [42] want to find out about individual differences in how responders respond to unfair offers. They want to see how apologies by proposers who make unfair offers may make recipients accept the offers, according to individual differences with respect to how stressed they become in cases of uncertainty. In the second study, Zheng, Van Dijke, Leunissen, Giurge, and De Cremer [43] also ask how apologies may fail, but as a result of power asymmetry. They hypothesize, and confirm, that if an apology is expressed by high-power people, victims are more likely to dismiss the apology and treat it cynically than when an apology is expressed by low-power people.

### Kantian theory contra utilitarian theory

Interestingly, while there can be degrees of insincerity, there are no degrees of sincerity. If one reveals one’s true intentions, one is sincere. One cannot be graded as either excessively or moderately sincere. Kant noted this fact. To wit, Kant celebrated sincerity, which he calls “good will”, precisely because there cannot be “too much” sincerity. Kant stated at the outset of his major work on moral philosophy, *Groundwork of the Metaphysics of Morals* (2^ND^ ed., 1786): “It is impossible to think of anything at all in the world, or indeed even beyond it, that could be taken to be good without limitation, except a GOOD WILL [GUTER WILLE].”

Granted that this is a brief characterization of Kant’s ethics. There is a wider meaning to Kant’s goodwill: moral agency stands outside nature and hence can only be good [7]. Also, Kant’s goodwill need not entail “perfect duty”, the area of formal or juridical obligation. Kant’s goodwill also entails “imperfect duty”: the actor may vary the act in light of his or her inclinations (what economists call “preferences”) and circumstances (what economists call “constraints”) [44].

For brevity, and ignoring these details in Kant’s ehtical theory, we call the position that stresses sincerity “Kantian theory”:

Kantian theory: For people to judge a deed agreeable, what matters is the sincerity behind it. The consequence in terms of welfare either do not matter (early Kant) or, if they matter, they matter less than sincerity (late Kant).

In contrast, we call the standard economist position “utilitarian theory”:

Utilitarian theory: For people to judge that a deed is agreeable, what matters is the consequences of the act in terms of its welfare impact.

Our experiment is inspired by the hypothesis that sincerity matters. We ask: Does insincerity aversion exist? Do people respond differently if they suspect others are using a Good Act instrumentally, i.e., as a moral license? In practice, moral licensing is difficult to ascertain, since it requires knowing someone’s intentions (hence the disagreement between Christopher Hitchens and Mother Theresa described above). As experimenters, we should be extremely cautious about claiming to know the intentions of participants in an experimental setting. The innovation of our experimental design is that we do not need to do so. Instead, we leave the matter of discerning intentions to the experimental participants themselves: one set of participants makes decisions that might be consistent with moral licensing, and we observe how another set of participants reacts.

## Experiment

### Research question

The focus of our experiment is on the ultimatum bargaining game [45], henceforth UG. In this game, there is a “cake” of ten dollars available to be split between two players, a proposer and a responder. The proposer chooses an allocation, which the responder can accept (so that it is implemented) or reject (so that both receive nothing). While equal and near-equal splits are feasible, the standard-theory prediction is that the proposer will demand all or nearly all of the cake, anticipating that the responder will accept any positive offer as being better than getting nothing. By contrast, a large and well-known experimental literature has found that, in practice, proposers offer to keep for themselves about 50–80 percent of the cake, while responders frequently reject small but positive offers (see [46] and [47], pp. 48–83]. Brañas-Garza, Cobo-Reyes, and Domínguez [48], though, show that in some field experiments in close-knit communities where people knew each other, the few responders who received zero offers, accepted them. When interviewed, these few responders used a value norm: if the proposer offered only zero to us, the proposer must have needed the whole cake. This exception, indeed, confirms the larger literature. Responders are usually intolerant of unfair offers, unless the responders know that many of the proposers are in great need.

The two stylized facts that (a) “fair” outcomes are possible but “unfair” behavior is likely to be seen, and (b) scope exists for punishing unfair behavior (i.e., rejecting the proposal) make the UG a useful base for our study. Because proposers face a monetary incentive to behave unfairly, it is likely that many will do so. Some may genuinely regret this decision and will apologize (sincerely) if given the option. But others may not be genuine or authentic in their apology; they may have another, instrumental motive for apologizing: convincing the responder to accept their proposal instead of rejecting it.

As experimenters, we cannot tell if the intention behind a proposer’s apology is authentic or insincere. Rather, we focus on how the responders view the matter—what we want to gauge is whether the sincerity of proposers matters at all to the responders. If it does not matter, responders should not vary their decisions in response to the treatment in our experiment. Our experiment varies the treatment by changing the possibility and form of apologies in a way that makes it likely that participants in the experiment assess sincerity differently across treatments. We want to ascertain if responders care whether the apology is instrumental rather than sincere:

Research question: Does sincerity matter? Do spectators and victims exhibit insincerity aversion, i.e., are they ready to punish others if they suspect that those others behaved insincerely, even when such punishment has no strategic effect or is costly to the punishers?

There is a growing literature on apology messages in experimental psychology as mentioned above. But apology has gained little attention in the economics literaturet. There are some exceptions, though. Bénabou and Tirole [49] show how that if a good deed such an apology is executed instrumentally, i.e., insincerely, it becomes ineffective, or may even backfire. The psychological literature focuses on whether the apology helps to repair damaged trust and restore cooperation [50, 51]; the neural correlates of non-cooperative choices and forgiveness [52]; the healing effects of apology [53–56]; whether apology works in medical malpractice [57]; and whether apology is effective only if it is a costly signal that separates the serious from the non-serious and, further, whether the issuer of the apology may reap a benefit from the apology [58, 59]. While the focus of these studies is mostly on the redeeming effects of apology with respect to trust and cooperation, the focus of this paper is on whether responders care about the sincerity of the apology at all.

### Experimental design

Our experiment involves three treatments. Our baseline treatment is a standard one-shot UG. The proposer makes an offer of any whole-dollar amount between 0 and 10 inclusive to the responder, who observes the offer and chooses to accept (thus receiving the amount offered, with the proposer keeping the remainder) or reject (in which case each receives zero), after which the game ends.

Our other two treatments give the proposer the chance to apologize for the amount offered. Our intention is to vary the extent to which proposers’ apologies can be interpreted as insincere, in order to detect insincerity aversion. In our known-apology treatment, the proposer knows–at the time of choosing the offer–that she has the ability to send an apology (“Sorry for the proposal”) along with the offer. In this known-apology treatment, the responder also knows that the proposer had had the ability to send an apology at the time of making the offer—irrespective of whether the proposer had sent an apology. Put differently, the responder receives an apology (if the proposer had chosen it) or receives no apology (if otherwise) along with the offer and, in either case, is informed that the proposer became aware of the apology option concurrently with making the offer choice. The responder’s accept/reject decision is made after receiving all of this information.

In our known-apology (KA) treatment, it is likely for the responders to view the apology as insincere given that the issuer of the apology can derive some gain from the apology. This likelihood is consistent with Ho [59] who finds that the apology signal loses effectiveness as soon as the recipients find that the issuer may benefit from the apology. This likelihood is also consistent with the theory of Bénabou and Tirole [49] that good deeds (such as apologies) backfire if observers find that they stem from instrumental (i.e., insincere) motives.

In our unknown-apology (UA) treatment, the proposer has a similar opportunity to send an apology, but is not informed of this opportunity until after her offer choice is made. Some experimental economists view “surprise rule changes” as methodologically problematic, due to the potential loss of control over participants’ expectations. (That is, participants’ decisions may be affected by their conjectures about future additional surprise rule changes.) We acknowledge this criticism, but note that this technique is not unique to our study (see, for example, the “surprise message opportunity” in Chen and Houser’s [60] double-message treatment). Also, without such a “surprise”, it would be difficult to argue that proposers in this treatment and those in our baseline treatment faced similar conditions.

In any case, the responder receives the apology (or not, depending on the proposer’s choice) along with the offer and, in either case, is informed that the proposer became aware of the apology option only after the proposer had made the offer choice. The responder’s accept/reject decision is made after receiving all of this information.

In our unknown-apology treatment, the timing of information gives the responder *less reason* to suspect that the apology gesture was chosen instrumentally. This does not mean that the responder has *no reason* to suspect the intention behind the apology in this treatment. A responder may reason, “the proposer has proposed a low offer choice and is probably using the apology to manipulate me to accept the offer.” But this line of reasoning also exists in the known-apology treatment. Where the treatments differ is that in the unknown-apology treatment, the responder is certain that the possibility of apology could not have influenced the offer choice, whereas it might have been the case in the known-apology treatment. That is, the known-apology treatment involves an information structure that adds an extra reason for suspicion on top of the one that might exist in the unknown-apology information structure.

### Experimental procedures

Monash University Human Research Ethics Committee (MUHREC) has approved the project. We have obtained written consent forms from all participants. The participants were recruited using ORSEE [61] and they were primarily undergraduate students from a variety of disciplines, with a large fraction from economics and business-related disciplines. The experiment was conducted at Monash University’s experimental economics lab (MonLEE). No-one took part in more than one session; otherwise there were no exclusion criteria. The experiment was computerized, and programmed in z-Tree [62]. Participants were visually isolated, and were asked not to communicate other than via the program. No identifying information about participants (e.g., ID numbers) was provided to other participants.

**Fig 1:**
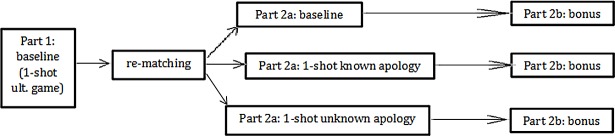
The structure of the experiment.

A session of the experiment comprised two parts (Fig 1). Part 1 was always a single round of the baseline (i.e., a standard UG). While behavior in Part 1 is not the primary concern of our study, we will use some Part-1 decisions as controls for unobserved characteristics in our analysis of Part 2.

After Part 1, participants remained in the same roles (proposer or responder), but were re-matched and then played Part 2: a single round of a game that depended on the treatment (another baseline game, a known-apology game, or an unknown-apology game). After making the accept/reject decision in Part 2, each responder was informed that he had an opportunity to send his matched proposer a “bonus”: an extra whole-dollar amount between 0 and 5 inclusive, at no monetary cost to the responder.

Thus, there are two ways the responder can express disapproval at the proposer’s offer. Rejecting the proposal punishes the proposer, but is costly for the responder; hence we expect to see rejections only if disapproval is sufficiently strong. Choosing a small or even zero bonus also punishes the proposer, but is costless for the responder; if disapproval is present but not strong, we would expect to see punishment expressed here rather than in rejections. Clearly, it is important that responders do not know about the opportunity to punish via bonuses when making the accept/reject decisions; otherwise they might choose the less costly punishment even if they were willing to use the more costly one.

To note, the reason behind the rejection of low offers may go beyond punishment, and indeed could be motivated by spitefulness or antisocial behavior [63–66]. Responders possessing such traits would be more likely across-the-board to reject low or even moderate offers. However, since these traits are likely to be individual-specific, we do not expect their effects to differ across our treatments. By the same token, it is possible that inequity aversion might hinder the decisions of responders to award bonuses; e.g., following an accepted offer of 5, the responder may prefer a zero bonus and resulting (5, 5) payoff pair to a positive bonus that would make the payoffs unequal. However, Capraro, Smyth, Mylona, and Niblo [67] found that a majority of their participants were “benevolent”–willing to incur a positive cost to increase another’s payoff beyond their own. If their result is general, it suggests that inequity aversion should not be a significant concern in our bonus stage, where increasing the proposer’s payoff is costless.

At the beginning of each session, participants were seated and given written instructions for Part 1; these were also read aloud by the experimenter (protocols: http://dx.doi.org/10.17504/protocols.io.tutenwn). There was no instructions quiz, but participants could ask clarifying questions at any point during the session; any such questions were answered privately. Instructions for Part 2 were presented only on computer screens. Participants received full feedback at the end of each part regarding their own outcome (offer, response and payoffs, and where relevant, apology and/or bonus), and received no information about other participant pairs’ decisions. Participants were paid, in cash, the sum of their earnings from both parts of this experiment.

The payments were in Australian dollars; at the time of the experiment, 1 AUD was worth roughly 75 US cents. Participants additionally received a $10 participation fee, and had the opportunity to take part in another experiment (involving a dictator game) immediately after this one. Participants received no information about this later experiment until the experiment discussed in this paper had concluded, and no other experiment took place before the one discussed here.

### Null hypotheses

Given the research question, we state the null hypotheses concerning the responders only, followed by a discussion of plausible alternative hypotheses corresponding to each one. Note, these null hypotheses are additionally the predictions of the utilitarian hypotheses (H^U^) since the utilitarian view assumes that individuals ultimately do not care about sincerity. That is, given the utilitarian assumption that responders only care about consequences, the likelihood of sincerity or insincerity of proposers should not matter:

H1a: Conditional on the offer, responders’ likelihood of accepting in the known-apology treatment will be equal to that in the baseline.
H1b: Conditional on the offer, responders’ likelihood of accepting in the unknown-apology treatment will be equal to that in the baseline.
H1c: Conditional on the offer, responders’ likelihood of accepting in the known-apology treatment will be equal to that in the unknown-apology treatment.
H2a: Conditional on the offer, responders’ bonus choices in the known-apology treatment will be equal to that in the baseline.
H2b: Conditional on the offer, responders’ bonus choices in the unknown-apology treatment will be equal to that in the baseline.
H2c: Conditional on the offer, responders’ bonus choices in the known-apology treatment will be equal to that in the unknown-apology treatment.

### Alternative hypotheses

The Kantian hypotheses (H^K^) make contrary predictions to our null hypotheses since the Kantian view assumes that individuals care about sincerity. Note, though, the Kantian view implies no difference with regard to H1b and H2b, where sincerity is irrelevant:

H^K^1a: Conditional on the offer, responders’ likelihood of accepting in the known-apology treatment will be lower than that in the baseline.
H^K^2a: Conditional on the offer, responders’ bonus choices in the known-apology treatment will lower than that in the baseline.
H^K^1c: Conditional on the offer, responders’ likelihood of accepting in the known-apology treatment will be lower than that in the unknown-apology treatment.
H^K^2c: Conditional on the offer, responders’ bonus choices in the known-apology treatment will be lower than that in the unknown-apology treatment.

This is the case because responders in the known-apology treatment make their accept/reject decision knowing that the proposers made their offers while knowing about the possibility of apologizing. That is, there is a greater possibility for the responders to interpret the apology as insincere–used instrumentally by the proposers–than in the unknown-apology treatment. While responders will not punish those who did not use the apology, we hypothesize that they will punish those who used it.

## Results

We conducted 16 sessions. Some large sessions were partitioned into smaller “matching groups” that were closed with respect to interaction; there were a total of 7, 9 and 7 matching groups in the baseline, known-apology and unknown-apology treatments respectively, with 100, 110 and 108 participants. Four additional participants had to be dropped from the data-set, as a programming error had put them into two matching groups of size two, ensuring that they were matched to the same opponent in both rounds. Thus, a total of 322 participants took part in the experiment, while our analysis is based on 318 participants.

Table 1 shows results from Part 1, when participants played a standard UG without the treatment. Offers averaged $4.40 and were accepted 87 percent of the time, with acceptances more likely as the offer increased. These results do not vary substantially by treatment, and are typical within the UG literature.

**Table 1:** Offer and acceptance frequencies from Part 1.

Offer ($)	0–1	2	3	4	5	6–10
Frequency chosen	0.013	0.088	0.132	0.233	0.421	0.113
Frequency accepted	0.000	0.357	0.762	0.865	1.000	1.000

Note: cake size = $10

The average offer in Part 1 was $4.38, $4.41 and $4.39 in the baseline, known-apology and unknown apology treatments respectively–almost identical. Also, a probit with Part-1 acceptance as the dependent variable and with a constant, the Part-1 offer, and treatment dummies as the independent variables yields insignificant coefficients for the treatment dummies, either separately or jointly. As an indication of power, if the entire data-set is replicated (thus doubling the sample size), the two treatment dummies become jointly significant. In order for the UA and KA indicators to be individually significant, the data-set would have to be replicated 4 and 8 times respectively; i.e., the data set would have to be 5 or 9 times (respectively) as large as it currently is. (Additional details are available from the authors upon request.)

### Proposer behavior

Table 2 shows proposer statistics from Part 2 by treatment. Average offers rise slightly from Part 1 to Part 2 in the baseline and fall slightly in the other two treatments, though these differences are either insignificant or marginally significant (two-sided Wilcoxon signed-ranks test, matching-group-level data, p ≈ 0.062 in the baseline; p ≈ 0.078 in the KA treatment, p > 0.20 in the UA treatment). Neither the changes nor the offers themselves are significantly different across treatments (two-sided Kruskal-Wallis test, matching-group-level data, p > 0.20 for both mean offers and mean changes in offer from Part 1). Hence the availability of apologies does not significantly affect the kinds of offers proposers make (though the direction of the effect is the one implied by proposers attempting to use moral licensing).

**Table 2:** Proposer statistics from Part 2.

Treatment:	Baseline	Known-apology	Unknown-apology
Mean offer ($)	4.680	4.018	4.074
Mean change in offer from Part 1 ($)	+0.300	−0.400	−0.315
Frequency of apology–overall	—	0.491	0.444
Frequency of apology–given offer 0–2	—	1.000	0.636
Frequency of apology–given offer 3	—	0.643	0.625
Frequency of apology–given offer 4	—	0.364	0.444
Frequency of apology–given offer 5	—	0.167	0.062
Frequency of apology–given offer 6–10	—	0.800	0.700

Table 2 suggests that the use of apologies is correlated with proposer offers, since offers are lower by about 8 percent of the cake (in the KA treatment) or 4 percent of the cake (in the UA treatment) when accompanied by an apology than without one. However, these differences are not significant in either treatment individually (Wilcoxon signed-ranks test, p > 0.20), and jointly the difference is only weakly significant (p ≈ 0.091). Overall, apologies are made just under half the time in both KA and UA treatments, with no significant differences between them (robust rank-order test, p > 0.20).

One odd aspect of the data visible in Table 2 is that apologies tend to accompany very high offers (more than half of the cake size) as well as low ones. Since there seems to have been little unusual in the history of play for those participants apologising for high offers (all of them had made an offer of either 4 or 5 in Part 1, and all of these offers had been accepted), we speculate that these participants are unusual in whatever ways that participants in ordinary ultimatum games making large offers are unusual.

We next use regressions to examine more closely the factors affecting proposer behavior. Model 1 is a Tobit with the proposer’s Part-2 offer as the dependent variable, and Model 2 is a probit with the proposer’s Part-2 apology as the dependent variable (hence Model 2 drops the baseline-treatment observations). The explanatory variables in each model are as shown in Table 3, plus a constant term and controls for the day, start time and number of participants in each experimental session. The models were estimated in Stata (version 11) and included robust standard errors clustered at the matching-group level.

**Table 3:** Proposer estimation results–Marginal effects (ME), standard errors in parentheses.

	[1]	[2]
Dependent variable:	Offer in Part 2	Apology in Part 2
Known-apology (KA) treatment	−0.736*** (0.198)	−0.135 (0.096)
Unknown-apology (UA) treatment	−0.608 (0.460)	
*Treatment effect (KA vs*. *UA)*?	*p > 0*.*20*	*p ≈ 0*.*16*
Part-2 offer (unconditional ME)		−0.070*** (0.023)
(ME, KA treatment)		−0.098*** (0.037)
(ME, UA treatment)		−0.032 (0.022)
*Treatment effect (KA vs*. *UA)*?		*p ≈ 0*.*13*
Offer in Part 1	0.294* (0.171)	
Low offer (0–2) accepted in Part 1	−1.753*** (0.416)	
High offer (4+) rejected in Part 1	−0.034 (0.314)	
Additional controls	Session day, session time, number of participants in session	
N	159	109
|ln(L)|	275.64	68.53

*, ***: Significantly different from zero at 10, 1 percent level.

Table 3 shows the results. From Model 1, the marginal effect of the KA treatment indicator is negative and significant, implying that offers are significantly lower in the KA treatment than in the baseline treatment, consistent with proposers attempting to use moral licensing. The marginal effect of the UA indicator, while also negative, is insignificant.

Result 1: Offers are lower in the known-apology treatment than in the baseline.
Result 2: Offers are not significantly different between the unknown-apology treatment and the baseline.

From Model 2, the negative marginal effect for the KA treatment indicator implies apologies are less likely in that treatment (other things equal), but this difference is not significant. However, the negative marginal effects of the Part-2 offer variable in both KA and UA treatments indicate negative correlations between the offer and apology (apologies are more likely following low offers) in both of these treatments–consistent with Table 2. The effect is larger and statistically significant in the case of the KA treatment, and smaller and insignificant for the UA treatment, though the difference between treatments just misses significance.

**Fig 2:**
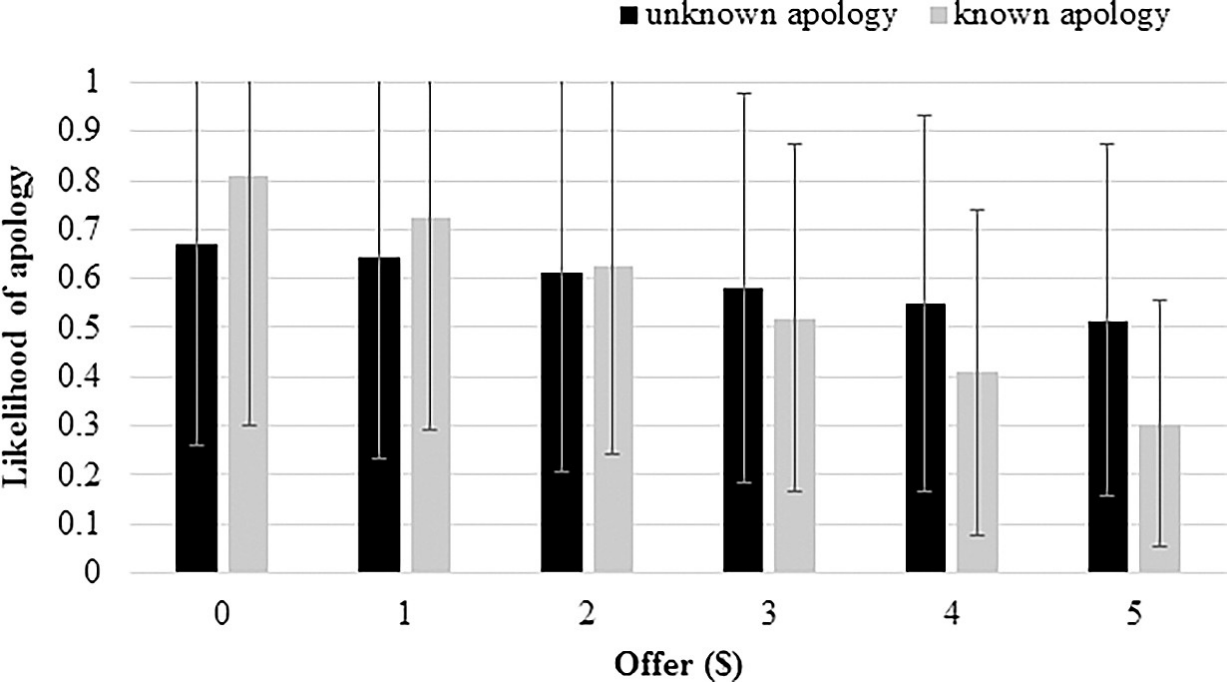
Estimated likelihood of apology based on Model 2 in Table 3.

Fig 2 illustrates these correlations. Fig 2 displays the predicted likelihood of an apology conditional on various offers, based on the results of model 2 (point estimates, with 95-percent confidence intervals shown by error bars). Though the wide confidence intervals mean that differences in predictions between the treatments are insignificant, Fig 2 suggests that for low offers, apologies may be ***more*** likely in the KA treatment, while for higher offers, apologies may be ***less*** likely in the KA treatment.

To the extent that the relationship between offer and apology is stronger in our KA treatment than in our UA treatment, one plausible explanation is as follows. Consider the participants in our KA treatment counterfactually undergoing the procedures of our UA treatment, with apologies not known to be available until after the offer was made. It is reasonable to expect that the distribution of offers would have been comparable to that in the baseline treatment, and the correlation between offer and apology would have been like that in the UA treatment. Moving from that counterfactual to the case where apologies were known to be available at the time offers were chosen (i.e., the actual procedures this group underwent), we would expect that some proposers would opportunistically lower their offers and include an apology. If so, this would result in low offers more often accompanied by apologies in the KA treatment than in the UA treatment, while the frequency of apologies accompanying higher offers would be similar between the two treatments. This is consistent with what we see in Fig 2.

The results concerning the proposers’ behavior suggest that while proposers do tend to use apologies when making a low offer, there is little evidence that they do so in an opportunistic way: they are not significantly more likely to use apologies when they know *ex-ante* of their availability, nor do they offer significantly less when they have this information.

### Responder behavior

Table 4 shows descriptive statistics for responders in Part 2: their frequency of acceptance and average bonus conditional on the offer, the treatment, and whether the proposer chose to make an apology. Drawing conclusions based on these descriptive statistics is complicated by small numbers of observations in many of the contingencies (those in bold-face represent sample sizes of more than 5). For the most part, we observe the usual positive relationship between the amount offered and both the likelihood of acceptance and average bonuses, with the possible exception of very large offers (more than half of the surplus) which appear to be accepted less often and garner lower bonuses compared to offers of equal splits. Indeed, other studies have observed rejections of overly generous offers, perhaps due to extreme aversion to advantageous inequity [68]. However, it is difficult to discern differences across treatments or according to whether an apology was given.

**Table 4:** Responder acceptance frequencies and average bonuses, Part 2.

Offer ($)	0–2	3	4	5	6–10
*Acceptance frequency*					
Baseline	0.00	**0.67**	**0.90**	**1.00**	**0.83**
Known apology | no apology	—	0.80	**1.00**	**1.00**	1.00
Known apology | apology	**0.57**	**0.56**	1.00	1.00	0.75
Unknown apology | no apology	0.50	1.00	0.80	**1.00**	0.67
Unknown apology | apology	**0.29**	0.80	1.00	1.00	**1.00**
*Average bonus*					
Baseline	0.00	**1.22**	**2.90**	**3.71**	**2.83**
Known apology | no apology	—	4.20	**3.29**	**4.33**	2.00
Known apology | apology	**1.86**	**2.11**	3.25	3.33	3.50
Unknown apology | no apology	1.50	2.00	0.20	**3.67**	2.00
Unknown apology | apology	**2.71**	2.20	0.25	5.00	**3.43**

Note: cake size = $10. Bold entries comprise more than 5 observations.

In order to separate the effects of the treatment, offer, and apology from each other and from other “nuisance” variables, we use regressions. We use either a probit model with an acceptance in Part 2 as the dependent variable (referred to as models 3–4 below), or a Tobit model with the bonus as the dependent variable (models 5–8). The right-hand-side variables include a constant, the amount offered in Part 2, indicators for the KA and UA treatments, and indicators for each of those treatments combined with an apology being made, as well as the session controls used for the proposer regressions in Table 3. In addition to those regressions reported here, we ran another set that included the square of the Part-2 offer, to allow for the non-monotonic effect suggested by Table 4. The offer-squared variable was negative and significant in the analogues to models 3 and 4 (consistent with a concave effect on acceptances), while it was never significant in the analogues to models 5–8 (suggesting a linear effect on bonuses). Including the offer-squared variable did not affect any of the other results reported in Table 5; hence we omit these results to save space.

**Table 5:** Responder estimation results–Marginal effects and standard errors (N = 159).

	[3]	[4]	[5]	[6]	[7]	[8]
Depend. variable:	Acceptance in Part 2		Bonus in Part 2			
Part-2 offer	0.070*** (0.021)	0.064*** (0.020)	0.807*** (0.292)	0.740*** (0.278)	0.399 (0.255)	0.372 (0.254)
KA treatment, ***no apology***	0.096 (0.092)	0.080 (0.094)	3.754*** (1.059)	3.538*** (1.117)	3.543*** (0.955)	3.404*** (1.014)
UA treatment, ***no apology***	0.037 (0.103)	0.064 (0.101)	−1.000 (1.449)	−0.646 (1.394)	−0.930 (1.527)	−0.654 (1.496)
*Signif*. *difference (KA vs*. *UA)*?	*p > 0*.*20*	*p > 0*.*20*	***p ≈ 0*.*002***	***p ≈ 0*.*005***	***p ≈ 0*.*003***	***p ≈ 0*.*007***
KA treatment, ***apology***	−0.172 (0.134)	−0.197 (0.125)	0.433 (1.253)	0.052 (1.258)	1.101 (1.094)	0.805 (1.132)
UA treatment, ***apology***	−0.016 (0.068)	−0.037 (0.064)	−0.405 (1.173)	−0.541 (1.171)	0.001 (1.157)	−0.097 (1.155)
*Signif*. *Difference (KA vs*. *UA)*?	*p > 0*.*20*	*p > 0*.*20*	*p > 0*.*20*	*p > 0*.*20*	*p > 0*.*20*	*p > 0*.*20*
Rejected Part-1 offer of 4+		−0.348*** (0.098)		−4.796 (2.856)		−3.746 (2.754)
Part-2 acceptance					4.737*** (1.579)	4.548*** (1.543)
Additional controls	Session day, session time, number of participants in session (all 6 models)					
|ln(L)|	56.99	55.64	247.76	246.47	241.50	240.71

***: Significantly different from zero at 5, 1 percent level.

In models 3 and 5, no other variables were included. In a second pair of models (models 4 and 6), we attempted to control for any potential strong inequity aversion by the responder, by including an indicator variable equal to one if the responder was offered 40 percent of the cake or more and rejected the offer. The remaining models (7 and 8) included the Part 2 accept decision itself; since this decision took place before responders were told there would be a bonus decision, we can consider the acceptance decision to be exogenous to the bonus decision. (Model 8 additionally uses the “Rejected Part-1 offer of 4+” variable used in models 4 and 6.) As with the proposer regressions, these models were estimated using Stata with standard errors clustered by matching group.

Table 5 shows that the results are mostly robust to specification. Between models with and without the “Rejected Part-1 offer of 4+” variable, there is little difference in the marginal effects of the other variables, though its own effects are negative as we would expect, and the effect on acceptances is significant. Also, the “Part-2 acceptance” variable is highly significant, with a positive value suggesting that acceptance and bonus choices are complements rather than substitutes. Comparison of models with and without this variable shows one notable difference (the effect of the Part-2 offer itself becomes weak and insignificant once the acceptance decision is added), but the treatment effects themselves are not substantially affected.

The marginal effect of the Part-2-offer variable is positive and significant whenever the “Part-2 acceptance” variable is not in the regression, confirming that higher Part-2 offers are associated with more frequent acceptance and higher bonuses (as Table 4 had suggested). Also, none of the UA treatment indicators–those for receiving an apology or those for not receiving one–are significant, suggesting that responders’ behavior (both acceptances and bonuses) is similar in the unknown-apology treatment to the baseline. (As an indication of power, the entire data-set would need to be 9 times its current size (i.e., replicated 8 times) in order for any of the twelve UA marginal effects in Table 5 to become significant.). Thus, our concern stated above–that responders might have become suspicious of the intention of proposers if they chose apology in the UA treatment–is not borne out in the data.

However, the KA treatment does matter, and it does so in a way that supports Kantian theory. Specifically, responders are more likely to reward an offer with a bonus when it is not accompanied by an apology compared to the baseline treatment, as shown by the positive and significant marginal for the KA-no-apology indicator in models 5–8. They are also more likely to accept a given offer when it is without an apology, but this latter difference is not significant. Responders are also more likely to accept and award bonuses when the offer ***is not*** accompanied by an apology compared to when the offer ***is*** accompanied by an apology, though the difference is only significant for bonuses (not shown in table, p-values of between 0.013 and 0.018 in models 5–8, versus 0.15 and 0.13 in models 3 and 4 which dealt with acceptances). While the effect on acceptances is not significantly different from the corresponding effect in the UA treatment (p > 0.20 in models 3–4), the effect on bonuses is significantly larger (p-values between 0.002 and 0.007 in models 5–8). That is, if we control for the offer amount, responders award lower bonuses to those who use an apology in the known-apology treatment than to those who use an apology in the unknown-apology treatment.

Result 3: In the known-apology treatment, responders award lower bonuses for a given offer when it is accompanied by an apology than without an apology, while there is no corresponding significant difference in acceptances.
Result 4: In the unknown-apology treatment, responders’ acceptance probabilities and bonuses for a given offer are not significantly different according to whether or not an apology accompanies the offer.

To summarize, while responders in the KA treatment do not punish proposers who use the apology by rejecting their offer, the responders do punish those proposers indirectly. Responders give greater bonuses to proposers who refrain from using the known apology than to proposers who use it, *ceteris paribus*. While insincerity aversion is not intense enough to entail an increase in costly punishment in our experiment, insincerity aversion does make costless punishment more likely. So, our results support Kantian theory: people care about sincerity. If people suspect that the actor is using the Good Act for an instrumental purpose–as moral licensing–they punish it if the cost of doing so is sufficiently low. But if people judge the apology to be sincere–as moral equilibrium–then they will not punish it.

## Conclusion

We did not find statistically significant evidence that proposers behave more opportunistically in the known-apology treatment compared to the unknown-apology treatment. However, after controlling for the money offered, proposers who did not use the apology in the known-apology treatment were rewarded more than those who did not use the apology in the unknown-apology treatment. That is, a substantial portion of the responders seemingly cared about sincerity and were ready to reward proposers who refrained from misusing knowingly available apology messages.

As noted in our experimental procedures section above, our participant pool is over-represented by economics and business-related disciplines. Many studies have shown that students drawn from such a participant pool behave in a more self-regarding (as opposed to other-regarding) way than the general population [69, 70]. So, the results of our study can be viewed as a conservative test of insincerity aversion. That is, in the general population, the phenomenon of insincerity aversion is likely to be more pronounced than what is found in our results.

This evidence of insincerity aversion supports the Kantian theory as a good description of human sentiments and choices. What matters is not only the consequence of an action; the sincerity behind an action, or lack thereof, also matters. People care so much about sincerity that they are ready to reward their counterparts if they judge them to be sincere, even where evidence for this sincerity is purely circumstantial. Namely, people are ready to reward their counterparts if they refrain from using a known-apology even though it seems not to have influenced the offer choice itself.

For future research, it is worth investigating if there are gender differences with respect to Kantian theory. Capraro and Sippel [71] made some headway by uncovering an asymmetry: women abide by moral duty (the deontological principle) more than men in personal relations, but this is reversed for impersonal relations. It would be fruitful to see if such an asymmetry also holds with respect to insincerity aversion.

Future research may also investigate why people become angry at, and even hateful of, hypocritical decision makers. People find it particularly egregious when religious figures and public moralists abuse others in secret while publicly signaling their virtue [72]. Likewise, some free and non-coercive exchanges in the market can elicit anger, even by third-party spectators, if one party to the exchange assures others of his or her trustworthiness by referring to things like philanthropic work, commitment to communal values, commitment to religion, or the virtue of his or her parents or spouse. The anger directed at Bernard Madoff, even by people untouched by his Ponzi scheme, illustrates such insincerity aversion—the fact that Madoff used his moral credentials and communal connections to intentionally deceive others is seen as particularly deplorable [73].

For future research, it is important to distinguish the explicit deception of others, popularly called “con man” acts, from self-deception, i.e., where the deceiver also deceives the self about his or her intentions. Is the cheating pattern arising from insincerity different if it arises from explicit deception alone compared to self-deception? Firstly, though, future research should uncover whether it is possible to empirically delineate cheating that arises from insincerity toward others, i.e., explicit deception, as opposed to cheating that arises from insincerity toward the self, i.e., self-deception.

The economics literature has already touched on a related phenomenon: price gouging. People usually assume the fairness norm when they enter into bargaining negotiations. The fairness norm explains why driving a “hard bargain” or practicing “price gouging” is usually regarded as insincere or exploitative [17, 18, 74, 75]. Also, when parties level unfair punishments on others, traders withhold cooperation even when it also means inflicting harm on themselves [76]. Likewise, when people make offers judged as “too little, too late,” responders usually withhold cooperation. The withdrawal of cooperation from the perceived exploiter who is involved in non-coercive exchange and hence has not violated anyone’s property rights can be interpreted as deserving insofar as the exploiter is perceived to be hypocritical, i.e., has pretended to act out of sincerity.

The relevance of sincerity may also explain why many people judge a warm-glow in charity giving as distasteful [77]. In charity giving, the benefactor is stating to the self, irrespective of whether the donation is made known publicly, the intention of helping others. But insofar as the giving is partly for another purpose, such as receiving vicarious pleasure or public applause, the act would be insincere.

While we are confident that our main finding is robust, we note that elsewhere, our reliance on statistical significance to support our results entails some unsatisfactory potential conclusions. For example, the significant negative marginal effect of the known-apology dummy in Table 3 (model 1) suggests that offers are lower in our known-apology treatment than in our baseline treatment. However, we also see in Table 3 that offers are not significantly different between the known- and unknown-apology treatments, nor between the latter and our baseline treatment. This suggests that the power of our experiment is less than ideal (i.e., a larger sample would have been useful). Similarly, some of the secondary results involving proposer behaviour were marginally significant (with p-values between 5 and 10 percent); a larger experiment could either strengthen those findings or reveal them to have been mere statistical noise.

We close by taking note of fruitful future research that can explore other variants of moral licensing. One variant is where an agent highlights the crookedness or immorality of the other in order to justify his or her own crooked actions. For example, gangsters usually justify their actions by claiming that policemen and politicians are corrupt. Similarly, in the geopolitical arena, history is littered with examples where aggressors justify their aggression insincerely: they claim that the victim lacks virtue, embodies evil, is barbaric, is anti-democratic, hates God, hates freedom, and so on. The issue of sincerity and insincerity aversion promises to open new vistas and fresh perspectives on old problems of human behavior.

## Supporting information

S1 File Data—Moral Licensing—UG exp. (PDF)

S2 File Instruction Protocols.pdf. Protocols: http://dx.doi.org/10.17504/protocols.io.tutenwn.
(PDF)
