# Supplementary material for: Moral licensing, instrumental apology and insincerity aversion: Taking Immanuel Kant to the lab
Source: PLoS One. 2018 Nov 8;13(11):e0206878. doi: 10.1371/journal.pone.0206878 (PMC6224065; doi:10.1371/journal.pone.0206878)
Supplement: S1 File — (PDF) [file pone.0206878.s001.pdf]

Session Period Subject Group Profit TotalProfit
Participate Demand Apology Response Payoff OppPayoff
Type TypeDraw GroupDraw SubsessionOrder Subsession
num_group rank_group TimeOKInformationOK
TimeOKIntroAndProposalOK Remainder TimeOKPhase1OK
TimeOKPhase2InstructionsOK TimeOKPhase2InstructionAOK
TimeOKInstructionAResponseOK exptoproposer
TimeOKPhase2ExtraGivingOK PropPayoff RespPayoff
TimeOKCalculatePayoffsAndShowOutcomeOK
TimeOKFeedbackInLastRoundOK TimeDemandIntroAndProposalOK
TimeResponsePhase1OK Treatment
1 1 1 2 7 7 0
3 -1 1 7 3 1 1062301 1030401
12 1 20 0.1 29 0 7 20
0 0 0 0 0 7 3 21
0 17 0 0
1 1 2 5 5 5 0
5 -1 1 5 5 0 1010702 1032202
2 1 20 0.25 29 45 5
0 - - - 0 - 5 5
24 - 0 24 0
1 1 3 6 5 5 0
5 -1 1 5 5 1 1075003 1045503
15 1 20 0.3 29 0 5
25 - - - 0 - 5 5
23 - 29 0 0
1 1 4 7 0 0 0
3 -1 0 0 0 0 1001404 1085404
1 1 20 0.35 29 40 7
0 - - - 0 - 0 0
19 - 0 25 0
1 1 5 1 0 0 0
2 -1 0 0 0 0 1036905 1009105
8 1 20 0.05 29 40 8
0 - - - 0 - 0 0
27 - 0 28 0
1 1 6 5 5 5 0
5 -1 1 5 5 1 1068706 1044006
14 1 20 0.25 29 0 5
21 - - - 0 - 5 5
3 - 24 0 0
1 1 7 1 0 0 0
2 -1 0 0 0 1 1037407 1025907
9 1 20 0.05 29 0 8
25 - - - 0 - 0 0
19 - 8 0 0
1 1 8 2 3 3 0
3 -1 1 3 7 0 1023808 1015008
6 1 20 0.1 29 41 7
0 - - - 0 - 7 3
22 - 0 17 0
1 1 9 3 5 5 0
5 -1 1 5 5 0 1032109 1017909
7 1 20 0.15 29 44 5

0	–	–	–	0	–	5	5
25	–	0	24	0			
1	1	10	8	5	5	0	
5	-1	1	5	5	1	1067910	1094110
13	1	20	0.4	29	0	5	
27	–	–	–	0	–	5	5
26	–	37	0	0			
1	1	11	4	5	5	0	
5	-1	1	5	5	1	1095211	1036611
16	1	20	0.2	29	0	5	
25	–	–	–	0	–	5	5
25	–	28	0	0			
1	1	12	4	5	5	0	
5	-1	1	5	5	0	1014412	1021512
4	1	20	0.2	29	45	5	
0	–	–	–	0	–	5	5
15	–	0	24	0			
1	1	13	8	5	5	0	
5	-1	1	5	5	0	1011413	1098513
3	1	20	0.4	29	42	5	
0	–	–	–	0	–	5	5
16	–	0	27	0			
1	1	14	3	5	5	0	
5	-1	1	5	5	1	1051214	1032714
10	1	20	0.15	29	0	5	
22	–	–	–	0	–	5	5
23	–	31	0	0			
1	1	15	6	5	5	0	
5	-1	1	5	5	0	1022815	1058115
5	1	20	0.3	29	39	5	
0	–	–	–	0	–	5	5
18	–	0	-16	0			
1	1	16	7	0	0	0	
3	-1	0	0	0	1	1055316	1081716
11	1	20	0.35	29	0	7	
27	–	–	–	0	–	0	0
20	–	7	0	0			
1	1	17	9	4	4	0	
6	-1	1	4	6	1	2045217	2010317
20	2	20	0.45	29	0	4	
20	–	–	–	0	–	4	6
23	–	21	0	0			
1	1	18	11	5	5	0	
5	-1	1	5	5	0	2032618	2074818
19	2	20	0.55	29	45	5	
0	–	–	–	0	–	5	5
24	–	0	25	0			
1	1	19	10	5	5	0	
5	-1	1	5	5	1	2086019	2080719
22	2	20	0.5	29	0	5	
27	–	–	–	0	–	5	5
24	–	36	0	0			
1	1	20	10	5	5	0	
5	-1	1	5	5	0	2002120	2066220

17	2	20	0.5	29	45	5	
0	–	–	–	0	–	5	5
13	–	0	24	0			
1	1	21	9	6	6	0	
6	-1	1	6	4	0	2013821	2031221
18	2	20	0.45	29	43	4	
0	–	–	–	0	–	4	6
24	–	0	18	0			
1	1	22	11	5	5	0	
5	-1	1	5	5	1	2085722	2094622
21	2	20	0.55	29	0	5	
23	–	–	–	0	–	5	5
20	–	32	0	0			
1	2	1	4	7	14	1	
3	-1	1	7	3	1	1062301	1024301
12	1	20	0.2	–	–	7	–
26	0	37	0	-9	7	3	22
18	–	–	0				
1	2	2	7	6	11	1	
6	-1	1	6	7	0	1010702	1063502
2	1	20	0.35	–	–	4	–
26	99999	0	3	0	4	6	27
18	–	–	0				
1	2	3	6	6	11	1	
5	-1	1	6	5	1	1075003	1041303
15	1	20	0.3	–	–	5	–
26	0	39	1	15	5	5	24
19	–	–	0				
1	2	4	2	5	5	1	
5	-1	1	5	10	0	1001404	1026904
1	1	20	0.1	–	–	5	–
26	99999	0	5	0	5	5	21
19	–	–	0				
1	2	5	6	5	5	1	
5	-1	1	5	6	0	1036905	1051605
8	1	20	0.3	–	–	5	–
26	99999	0	1	0	5	5	22
20	–	–	0				
1	2	6	7	7	12	1	
6	-1	1	7	6	1	1068706	1051806
14	1	20	0.35	–	–	4	–
26	0	43	3	6	4	6	7
12	–	–	0				
1	2	7	8	8	8	1	
4	-1	1	8	4	1	1037407	1058307
9	1	20	0.4	–	–	6	–
26	0	40	2	8	6	4	24
13	–	–	0				
1	2	8	8	4	7	1	
4	-1	1	4	8	0	1023808	1086708
6	1	20	0.4	–	–	6	–
26	99999	0	2	0	6	4	29
13	–	–	0				
1	2	9	3	5	10	1	

5	-1	1	5	8	0	1032109	1026909
7	1	20	0.15	-	-	5	-
26	99999	0	3	0	5	5	27
20	-	-	0				
1	2	10	3	8	13	1	
5	-1	1	8	5	1	1067910	1020810
13	1	20	0.15	-	-	5	-
26	0	41	3	17	5	5	26
19	-	-	0				
1	2	11	2	10	15	1	
5	-1	1	10	5	1	1095211	1016211
16	1	20	0.1	-	-	5	-
26	0	44	5	27	5	5	29
19	-	-	0				
1	2	12	4	3	8	1	
3	-1	1	3	7	0	1014412	1031012
4	1	20	0.2	-	-	7	-
26	99999	0	0	0	7	3	21
16	-	-	0				
1	2	13	1	3	8	1	
3	-1	1	3	8	0	1011413	1017713
3	1	20	0.05	-	-	7	-
26	99999	0	1	0	7	3	24
99999	-	-	0				
1	2	14	1	8	13	1	
3	-1	1	8	3	1	1051214	1003014
10	1	20	0.05	-	-	7	-
26	0	41	1	21	7	3	22
13	-	-	0				
1	2	15	5	0	5	1	
4	-1	0	0	0	0	1022815	1044415
5	1	20	0.25	-	-	6	-
26	42	0	0	0	0	0	21
17	-	-	0				
1	2	16	5	0	0	1	
4	-1	0	0	0	1	1055316	1034116
11	1	20	0.25	-	-	6	-
26	0	41	0	20	0	0	27
18	-	-	0				
1	2	17	9	10	14	1	
5	-1	1	10	5	1	2045217	2009917
20	2	20	0.45	-	-	5	-
26	0	40	5	15	5	5	27
10	-	-	0				
1	2	18	10	5	10	1	
5	-1	1	5	10	0	2032618	2059618
19	2	20	0.5	-	-	5	-
26	99999	0	5	0	5	5	27
20	-	-	0				
1	2	19	11	9	14	1	
6	-1	1	9	6	1	2086019	2079719
22	2	20	0.55	-	-	4	-
26	0	44	5	15	4	6	19
20	-	-	0				

1	2	20	9	5	10	1	
5	-1	1	5	10	0	2002120	2006420
17	2	20	0.45	–	–	5	–
26	45	0	5	0	5	5	24
19	–	–	0				
1	2	21	11	6	12	1	
6	-1	1	6	9	0	2013821	2071421
18	2	20	0.55	–	–	4	–
26	43	0	5	0	4	6	27
20	–	–	0				
1	2	22	10	10	15	1	
5	-1	1	10	5	1	2085722	2019322
21	2	20	0.5	–	–	5	–
26	0	43	5	17	5	5	25
18	–	–	0				
2	1	23	3	5	5	0	
5	-1	1	5	5	1	1085401	1023901
14	1	22	0.136363636		30	0	5
99999	0	0	0	0	0	5	5
99999	0	99999	0	0			
2	1	24	6	5	5	0	
5	-1	1	5	5	1	1082102	1048002
13	1	22	0.272727273		30	0	5
99999	–	–	–	0	–	5	5
99999	–	99999	0	0			
2	1	25	4	5	5	0	
5	-1	1	5	5	1	1056103	1037703
9	1	22	0.181818182		30	0	5
99999	–	–	–	0	–	5	5
99999	–	99999	0	0			
2	1	26	2	4	4	0	
6	-1	1	4	6	1	1080204	1020604
12	1	22	0.090909091		30	0	4
99999	–	–	–	0	–	4	6
99999	–	99999	0	0			
2	1	27	5	4	4	0	
6	-1	1	4	6	1	1076105	1043005
11	1	22	0.227272727		30	0	4
99999	–	–	–	0	–	4	6
99999	–	99999	0	0			
2	1	28	7	5	5	0	
5	-1	1	5	5	0	1002606	1057806
1	1	22	0.318181818		30	99999	5
0	–	–	–	0	–	5	5
99999	–	0	99999	0			
2	1	29	4	5	5	0	
5	-1	1	5	5	0	1011907	1028507
4	1	22	0.181818182		30	99999	5
0	–	–	–	0	–	5	5
99999	–	0	99999	0			
2	1	30	7	5	5	0	
5	-1	1	5	5	1	1094708	1074108
15	1	22	0.318181818		30	0	5
99999	–	–	–	0	–	5	5

99999	-	99999	0	0			
2	1	31	8	5	5	0	
5	-1	1	5	5	0	1007609	1098409
3	1	22	0.363636364		30	99999	5
0	-	-	-	0	-	5	5
99999	-	0	99999	0			
2	1	32	2	6	6	0	
6	-1	1	6	4	0	1050710	1022610
8	1	22	0.090909091		30	99999	4
0	-	-	-	0	-	4	6
99999	-	0	99999	0			
2	1	33	3	5	5	0	
5	-1	1	5	5	0	1031711	1023011
7	1	22	0.136363636		30	99999	5
0	-	-	-	0	-	5	5
99999	-	0	99999	0			
2	1	34	5	6	6	0	
6	-1	1	6	4	0	1024312	1032312
6	1	22	0.227272727		30	99999	4
0	-	-	-	0	-	4	6
99999	-	0	99999	0			
2	1	35	1	5	5	0	
5	-1	1	5	5	1	1097613	1006613
16	1	22	0.045454545		30	0	5
99999	-	-	-	0	-	5	5
99999	-	99999	0	0			
2	1	36	8	5	5	0	
5	-1	1	5	5	1	1058214	1084114
10	1	22	0.363636364		30	0	5
99999	-	-	-	0	-	5	5
99999	-	99999	0	0			
2	1	37	6	5	5	0	
5	-1	1	5	5	0	1007515	1037715
2	1	22	0.272727273		30	99999	5
0	-	-	-	0	-	5	5
99999	-	0	99999	0			
2	1	38	1	5	5	0	
5	-1	1	5	5	0	1020216	1001116
5	1	22	0.045454545		30	99999	5
0	-	-	-	0	-	5	5
99999	-	0	99999	0			
2	1	39	11	0	0	0	
4	-1	0	0	0	1	2090217	2079217
23	2	22	0.5	30	0	6	
99999	-	-	-	0	-	0	0
99999	-	99999	0	0			
2	1	40	10	6	6	0	
4	-1	1	6	4	1	2092118	2069718
24	2	22	0.454545455		30	0	6
99999	-	-	-	0	-	6	4
99999	-	99999	0	0			
2	1	41	9	5	5	0	
5	-1	1	5	5	0	2033619	2040619
19	2	22	0.409090909		30	99999	5

0	–	–	–	0	–	5	5
99999	–	0	99999	0			
2	1	42	11	0	0	0	
4	-1	0	0	0	0	2031820	2051720
18	2	22	0.5	30	99999	6	
0	–	–	–	0	–	0	0
99999	–	0	99999	0			
2	1	43	12	0	0	0	
1	-1	0	0	0	1	2074721	2079621
22	2	22	0.545454545		30	0	9
99999	–	–	–	0	–	0	0
99999	–	99999	0	0			
2	1	44	12	0	0	0	
1	-1	0	0	0	0	2024122	2065422
17	2	22	0.545454545		30	99999	9
0	–	–	–	0	–	0	0
99999	–	0	99999	0			
2	1	45	9	5	5	0	
5	-1	1	5	5	1	2060223	2059023
21	2	22	0.409090909		30	0	5
99999	–	–	–	0	–	5	5
99999	–	99999	0	0			
2	1	46	10	4	4	0	
4	-1	1	4	6	0	2037724	2040624
20	2	22	0.454545455		30	99999	6
0	–	–	–	0	–	6	4
99999	–	0	99999	0			
2	2	23	3	8	13	1	
5	-1	1	8	5	1	1085401	1049501
14	1	22	0.136363636		–	–	
5	–	99999	0	99999	3	99999	5
5	99999	99999	–	–	0		
2	2	24	7	10	15	1	
5	-1	1	10	5	1	1082102	1086302
13	1	22	0.318181818		–	–	
5	–	99999	0	99999	5	99999	5
5	99999	99999	–	–	0		
2	2	25	1	6	11	1	
5	-1	1	6	5	1	1056103	1026803
9	1	22	0.045454545		–	–	
5	–	99999	0	99999	1	99999	5
5	99999	99999	–	–	0		
2	2	26	4	9	13	1	
4	-1	1	9	4	1	1080204	1055804
12	1	22	0.181818182		–	–	
6	–	99999	0	99999	3	99999	6
4	99999	99999	–	–	0		
2	2	27	8	3	7	1	
7	-1	1	3	7	1	1076105	1090205
11	1	22	0.363636364		–	–	
3	–	99999	0	99999	0	99999	3
7	99999	99999	–	–	0		
2	2	28	3	5	10	1	
5	-1	1	5	8	0	1002606	1024606

1	1	22	0.136363636		–	–	
5	–	99999	99999	0	3	0	5
5	99999	99999	–	–	0		
2	2	29	5	5	10	1	
5	-1	1	5	6	0	1011907	1027707
4	1	22	0.227272727		–	–	
5	–	99999	99999	0	1	0	5
5	99999	99999	–	–	0		
2	2	30	2	9	14	1	
5	-1	1	9	5	1	1094708	1041908
15	1	22	0.090909091		–	–	
5	–	99999	0	99999	4	99999	5
5	99999	99999	–	–	0		
2	2	31	8	7	12	1	
7	-1	1	7	3	0	1007609	1083809
3	1	22	0.363636364		–	–	
3	–	99999	99999	0	0	0	3
7	99999	99999	–	–	0		
2	2	32	6	6	12	1	
6	-1	1	6	7	0	1050710	1035110
8	1	22	0.272727273		–	–	
4	–	99999	99999	0	3	0	4
6	99999	99999	–	–	0		
2	2	33	1	5	10	1	
5	-1	1	5	6	0	1031711	1000411
7	1	22	0.045454545		–	–	
5	–	99999	99999	0	1	0	5
5	99999	99999	–	–	0		
2	2	34	2	5	11	1	
5	-1	1	5	9	0	1024312	1020712
6	1	22	0.090909091		–	–	
5	–	99999	99999	0	4	0	5
5	99999	99999	–	–	0		
2	2	35	5	6	11	1	
5	-1	1	6	5	1	1097613	1055913
16	1	22	0.227272727		–	–	
5	–	99999	0	99999	1	99999	5
5	99999	99999	–	–	0		
2	2	36	6	7	12	1	
6	-1	1	7	6	1	1058214	1076014
10	1	22	0.272727273		–	–	
4	–	99999	0	99999	3	99999	4
6	99999	99999	–	–	0		
2	2	37	7	5	10	1	
5	-1	1	5	10	0	1007515	1072615
2	1	22	0.318181818		–	–	
5	–	99999	99999	0	5	0	5
5	99999	99999	–	–	0		
2	2	38	4	4	9	1	
4	-1	1	4	9	0	1020216	1025516
5	1	22	0.181818182		–	–	
6	–	99999	99999	0	3	0	6
4	99999	99999	–	–	0		
2	2	39	10	10	10	1	

5	-1	1	10	5	1	2090217	2051717
23	2	22	0.454545455		-	-	
5	-	99999	0	99999	5	99999	5
5	99999	99999	-	-	0		
2	2	40	9	0	6	1	
3	-1	0	0	0	1	2092118	2028418
24	2	22	0.409090909		-	-	
7	-	99999	0	99999	0	99999	0
0	99999	99999	-	-	0		
2	2	41	12	5	10	1	
5	-1	1	5	10	0	2033619	2057619
19	2	22	0.545454545		-	-	
5	-	99999	99999	0	5	0	5
5	99999	99999	-	-	0		
2	2	42	9	0	0	1	
3	-1	0	0	0	0	2031820	2005720
18	2	22	0.409090909		-	-	
7	-	99999	99999	0	0	0	0
0	99999	99999	-	-	0		
2	2	43	11	0	0	1	
2	-1	0	0	0	1	2074721	2081521
22	2	22	0.5	-	-	8	-
99999	0	99999	0	99999	0	0	99999
99999	-	-	0				
2	2	44	11	0	0	1	
2	-1	0	0	0	0	2024122	2041322
17	2	22	0.5	-	-	8	-
99999	99999	0	0	0	0	0	99999
99999	-	-	0				
2	2	45	12	10	15	1	
5	-1	1	10	5	1	2060223	2093623
21	2	22	0.545454545		-	-	
5	-	99999	0	99999	5	99999	5
5	99999	99999	-	-	0		
2	2	46	10	5	9	1	
5	-1	1	5	10	0	2037724	2019224
20	2	22	0.454545455		-	-	
5	-	99999	99999	0	5	0	5
5	99999	99999	-	-	0		
3	1	47	2	5	5	0	
5	-1	1	5	5	0	1005901	1038501
2	1	16	0.125	29	39	5	0
0	0	0	0	0	5	5	25
0	0	23	0				
3	1	48	1	6	6	0	
4	-1	1	6	4	1	1098802	1046502
16	1	16	0.0625	30	0	6	
24	-	-	-	0	-	6	4
26	-	27	0	0			
3	1	49	6	5	5	0	
5	-1	1	5	5	1	1050603	1083903
9	1	16	0.375	30	0	5	
25	-	-	-	0	-	5	5
24	-	22	0	0			

3	1	50	7	2	2	0	
2	-1	1	2	8	0	1038904	1091204
6	1	16	0.4375	30	45	8	
0	–	–	–	0	–	8	2
24	–	0	16	0			
3	1	51	2	5	5	0	
5	-1	1	5	5	1	1098405	1055405
15	1	16	0.125	30	0	5	
23	–	–	–	0	–	5	5
23	–	32	0	0			
3	1	52	8	5	5	0	
5	-1	1	5	5	1	1066106	1090906
10	1	16	0.5	30	0	5	
24	–	–	–	0	–	5	5
24	–	14	0	0			
3	1	53	5	5	5	0	
5	-1	1	5	5	1	1095907	1077907
14	1	16	0.3125	30	0	5	
26	–	–	–	0	–	5	5
25	–	37	0	0			
3	1	54	3	5	5	0	
5	-1	1	5	5	0	1046908	1046108
8	1	16	0.1875	29	36	5	
0	–	–	–	0	–	5	5
22	–	0	24	0			
3	1	55	8	5	5	0	
5	-1	1	5	5	0	1006309	1093709
3	1	16	0.5	24	43	5	
0	–	–	–	0	–	5	5
24	–	0	22	0			
3	1	56	4	3	3	0	
3	-1	1	3	7	0	1024710	1046910
4	1	16	0.25	30	44	7	
0	–	–	–	0	–	7	3
27	–	0	19	0			
3	1	57	3	5	5	0	
5	-1	1	5	5	1	1075911	1064711
11	1	16	0.1875	23	0	5	
21	–	–	–	0	–	5	5
24	–	-1	0	0			
3	1	58	5	5	5	0	
5	-1	1	5	5	0	1039912	1064512
7	1	16	0.3125	30	42	5	
0	–	–	–	0	–	5	5
23	–	0	27	0			
3	1	59	4	7	7	0	
3	-1	1	7	3	1	1084313	1073013
12	1	16	0.25	30	0	7	
21	–	–	–	0	–	7	3
28	–	38	0	0			
3	1	60	1	4	4	0	
4	-1	1	4	6	0	1001114	1026514
1	1	16	0.0625	30	39	6	
0	–	–	–	0	–	6	4

15	–	0	21	0			
3	1	61	6	5	5	0	
5	-1	1	5	5	0	1037415	1089915
5	1	16	0.375	26	34	5	
0	–	–	–	0	–	5	5
18	–	0	22	0			
3	1	62	7	8	8	0	
2	-1	1	8	2	1	1091616	1088516
13	1	16	0.4375	30	0	8	
24	–	–	–	0	–	8	2
22	–	41	0	0			
3	1	63	9	3	3	0	
3	-1	1	3	7	0	2036617	2017117
17	2	16	0.5625	30	43	7	
0	–	–	–	0	–	7	3
22	–	0	18	0			
3	1	64	9	7	7	0	
3	-1	1	7	3	1	2061718	2085918
18	2	16	0.5625	24	0	7	
24	–	–	–	0	–	7	3
22	–	4	0	0			
3	2	47	6	4	9	1	
4	-1	1	4	7	0	1005901	1082901
2	1	16	0.375	–	–	6	–
25	39	0	1	0	6	4	99999
99999	–	–	0				
3	2	48	6	7	13	1	
4	-1	1	7	4	1	1098802	1057402
16	1	16	0.375	–	–	6	–
25	0	99999	1	99999	6	4	99999
99999	–	–	0				
3	2	49	2	11	16	1	
4	-1	1	11	4	1	1050603	1008903
9	1	16	0.125	–	–	6	–
25	0	99999	5	99999	6	4	99999
99999	–	–	0				
3	2	50	8	3	5	1	
3	-1	1	3	7	0	1038904	1095804
6	1	16	0.5	–	–	7	–
25	99999	0	0	0	7	3	99999
99999	–	–	0				
3	2	51	7	5	10	1	
7	-1	1	5	7	1	1098405	1069405
15	1	16	0.4375	–	–	3	–
25	0	99999	2	99999	3	7	99999
99999	–	–	0				
3	2	52	5	9	14	1	
5	-1	1	9	5	1	1066106	1053606
10	1	16	0.3125	–	–	5	–
25	0	99999	4	99999	5	5	99999
99999	–	–	0				
3	2	53	1	10	15	1	
5	-1	1	10	5	1	1095907	1004007
14	1	16	0.0625	–	–	5	–

25	0	99999	5	99999	5	5	99999
99999	–	–	0				
3	2	54	4	0	5	1	
2	-1	0	0	0	0	1046908	1048908
8	1	16	0.25	–	–	8	–
25	43	0	0	0	0	0	99999
99999	–	–	0				
3	2	55	1	5	10	1	
5	-1	1	5	10	0	1006309	1013709
3	1	16	0.0625	–	–	5	–
24	45	0	5	0	5	5	99999
99999	–	–	0				
3	2	56	7	7	10	1	
7	-1	1	7	5	0	1024710	1085610
4	1	16	0.4375	–	–	3	–
28	45	0	2	0	3	7	99999
99999	–	–	0				
3	2	57	3	0	5	1	
6	-1	0	0	0	1	1075911	1013711
11	1	16	0.1875	–	–	4	–
24	0	99999	0	99999	0	0	99999
99999	–	–	0				
3	2	58	3	0	5	1	
6	-1	0	0	0	0	1039912	1045412
7	1	16	0.1875	–	–	4	–
23	45	0	0	0	0	0	99999
99999	–	–	0				
3	2	59	8	7	14	1	
3	-1	1	7	3	1	1084313	1079213
12	1	16	0.5	–	–	7	–
25	0	99999	0	99999	7	3	99999
99999	–	–	0				
3	2	60	5	5	9	1	
5	-1	1	5	9	0	1001114	1064214
1	1	16	0.3125	–	–	5	–
10	14	0	4	0	5	5	99999
99999	–	–	0				
3	2	61	2	4	9	1	
4	-1	1	4	11	0	1037415	1044715
5	1	16	0.125	–	–	6	–
25	44	0	5	0	6	4	99999
99999	–	–	0				
3	2	62	4	0	8	1	
2	-1	0	0	0	1	1091616	1029616
13	1	16	0.25	–	–	8	–
25	0	99999	0	99999	0	0	99999
99999	–	–	0				
3	2	63	9	2	5	1	
2	-1	1	2	8	0	2036617	2015517
17	2	16	0.5625	–	–	8	–
25	99999	0	0	0	8	2	99999
99999	–	–	0				
3	2	64	9	8	15	1	
2	-1	1	8	2	1	2061718	2097618

18	2	16	0.5625	–	–	8	–
25	0	99999	0	99999	8	2	99999
99999	–	–	0				
4	1	65	7	6	6	0	
4	-1	1	6	4	1	1059601	1091701
10	1	22	0.318181818		29	0	6
30	0	0		0	0	6	4
99999	0	36	0	1			
4	1	66	8	5	5	0	
5	-1	1	5	5	1	1073102	1096802
15	1	22	0.363636364		29	0	5
30	–	–		0	–	5	5
99999	–	38	0	1			
4	1	67	6	3	3	0	
3	-1	1	3	7	0	1055703	1055503
8	1	22	0.272727273		29	99999	7
0	–	–		0	–	7	3
99999	–	0	30	1			
4	1	68	5	5	5	0	
5	-1	1	5	5	1	1060604	1068404
11	1	22	0.227272727		29	0	5
30	–	–		0	–	5	5
99999	–	36	0	1			
4	1	69	7	4	4	0	
4	-1	1	4	6	0	1048205	1074005
7	1	22	0.318181818		29	40	6
0	–	–		0	–	6	4
99999	–	0	30	1			
4	1	70	8	5	5	0	
5	-1	1	5	5	0	1008906	1085706
1	1	22	0.363636364		29	41	5
0	–	–		0	–	5	5
99999	–	0	30	1			
4	1	71	6	7	7	0	
3	-1	1	7	3	1	1096507	1081307
16	1	22	0.272727273		29	0	7
30	–	–		0	–	7	3
99999	–	31	0	1			
4	1	72	1	5	5	0	
5	-1	1	5	5	0	1014808	1019708
2	1	22	0.045454545		29	39	5
0	–	–		0	–	5	5
99999	–	0	30	1			
4	1	73	4	5	5	0	
5	-1	1	5	5	1	1073009	1037009
14	1	22	0.181818182		29	0	5
30	–	–		0	–	5	5
99999	–	24	0	1			
4	1	74	3	3	3	0	
7	-1	1	3	7	1	1058510	1029810
9	1	22	0.136363636		29	0	3
30	–	–		0	–	3	7
99999	–	24	0	1			
4	1	75	1	5	5	0	

5	-1	1	5	5	1	1065011	1000111
12	1	22	0.045454545		29	0	5
30	-	-		0	-	5	5
99999	-	23	0	1			
4	1	76	3	7	7	0	
7	-1	1	7	3	0	1041012	1035012
6	1	22	0.136363636		29	45	3
0	-	-		0	-	3	7
99999	-	0	30	1			
4	1	77	4	5	5	0	
5	-1	1	5	5	0	1035213	1035213
4	1	22	0.181818182		29	37	5
0	-	-		0	-	5	5
99999	-	0	30	1			
4	1	78	5	5	5	0	
5	-1	1	5	5	0	1017114	1045014
3	1	22	0.227272727		26	39	5
0	-	-		0	-	5	5
99999	-	0	30	1			
4	1	79	2	7	7	0	
3	-1	1	7	3	1	1065115	1003415
13	1	22	0.090909091		29	0	7
30	-	-		0	-	7	3
99999	-	6	0	1			
4	1	80	2	3	3	0	
3	-1	1	3	7	0	1038616	1029716
5	1	22	0.090909091		29	41	7
0	-	-		0	-	7	3
99999	-	0	30	1			
4	1	81	9	10	10	0	
10	-1	1	10	0	0	2020517	2003317
19	2	22	0.409090909		29	40	0
0	-	-		0	-	0	10
99999	-	0	30	1			
4	1	82	10	7	7	0	
3	-1	1	7	3	1	2054318	2028518
23	2	22	0.454545455		29	0	7
30	-	-		0	-	7	3
99999	-	11	0	1			
4	1	83	9	0	0	0	
10	-1	1	0	10	1	2038519	2021419
21	2	22	0.409090909		29	0	0
30	-	-		0	-	0	10
99999	-	34	0	1			
4	1	84	12	4	4	0	
4	-1	1	4	6	0	2006420	2085820
18	2	22	0.545454545		29	45	6
0	-	-		0	-	6	4
99999	-	0	30	1			
4	1	85	11	5	5	0	
5	-1	1	5	5	1	2042021	2032421
22	2	22	0.5	29	0	5	
30	-	-		0	-	5	5
99999	-	22	0	1			

4	1	86	10	3	3	0	
3	-1	1	3	7	0	2005022	2036622
17	2	22	0.454545455		29	45	7
0	-	-		0	-	7	3
99999	-	0	30	1			
4	1	87	11	5	5	0	
5	-1	1	5	5	0	2037523	2070123
20	2	22	0.5	29	45	5	
0	-	-		0	-	5	5
99999	-	0	30	1			
4	1	88	12	6	6	0	
4	-1	1	6	4	1	2082924	2096624
24	2	22	0.545454545		29	0	6
30	-	-		0	-	6	4
99999	-	13	0	1			
4	2	65	7	6	12	1	4
0	1	6	4	1	1059601	1081601	10
1	22	0.318181818		-	-	6	-
99999	99999		0	99999	6	4	99999
99999	-	-	1				
4	2	66	6	11	16	1	4
1	1	11	4	1	1073102	1078802	15
1	22	0.272727273		-	-	6	-
99999	99999		5	99999	6	4	99999
99999	-	-	1				
4	2	67	6	4	7	1	4
1	1	4	11	0	1055703	1085503	8
1	22	0.272727273		-	-	6	-
99999	0		5	0	6	4	99999
99999	-	-	1				
4	2	68	1	10	15	1	5
0	1	10	5	1	1060604	1045904	11
1	22	0.045454545		-	-	5	-
99999	99999		5	99999	5	5	99999
99999	-	-	1				
4	2	69	8	3	7	1	3
1	1	3	12	0	1048205	1099805	7
1	22	0.363636364		-	-	7	-
99999	0		5	0	7	3	99999
99999	-	-	1				
4	2	70	3	0	5	1	3
0	0	0	1	0	1008906	1041806	1
1	22	0.136363636		-	-	7	-
99999	0		1	0	0	0	99999
99999	-	-	1				
4	2	71	3	1	8	1	3
0	0	1	0	1	1096507	1053407	16
1	22	0.136363636		-	-	7	-
99999	99999		1	99999	0	0	99999
99999	-	-	1				
4	2	72	4	5	10	1	5
0	1	5	10	0	1014808	1049808	2
1	22	0.181818182		-	-	5	-
99999	0		5	0	5	5	99999

99999	-	-	1				
4	2	73	5	10	15	1	5
0	1	10	5	1	1073009	1068209	14
1	22	0.227272727		-	-	5	-
99999	99999		5	99999	5	5	99999
99999	-	-	1				
4	2	74	4	10	13	1	5
0	1	10	5	1	1058510	1053410	9
1	22	0.181818182		-	-	5	-
99999	99999		5	99999	5	5	99999
99999	-	-	1				
4	2	75	2	8	13	1	3
1	1	8	3	1	1065011	1047311	12
1	22	0.090909091		-	-	7	-
99999	99999		1	99999	7	3	99999
99999	-	-	1				
4	2	76	5	5	12	1	5
0	1	5	10	0	1041012	1075312	6
1	22	0.227272727		-	-	5	-
99999	0		5	0	5	5	99999
99999	-	-	1				
4	2	77	7	4	9	1	4
0	1	4	6	0	1035213	1099213	4
1	22	0.318181818		-	-	6	-
99999	0		0	0	6	4	99999
99999	-	-	1				
4	2	78	1	5	10	1	5
0	1	5	10	0	1017114	1031914	3
1	22	0.045454545		-	-	5	-
99999	0		5	0	5	5	99999
99999	-	-	1				
4	2	79	8	12	19	1	3
1	1	12	3	1	1065115	1098115	13
1	22	0.363636364		-	-	7	-
99999	99999		5	99999	7	3	99999
99999	-	-	1				
4	2	80	2	3	6	1	3
1	1	3	8	0	1038616	1040916	5
1	22	0.090909091		-	-	7	-
99999	0		1	0	7	3	99999
99999	-	-	1				
4	2	81	11	3	13	1	3
1	1	3	12	0	2020517	2019117	19
2	22	0.5	-	-	7	-	99999
0		5	0	7	3	99999	
99999	-	-	1				
4	2	82	9	12	19	1	3
0	1	12	3	1	2054318	2009218	23
2	22	0.409090909		-	-	7	-
99999	99999		5	99999	7	3	99999
99999	-	-	1				
4	2	83	12	10	10	1	5
0	1	10	5	1	2038519	2051119	21
2	22	0.545454545		-	-	5	-

99999	99999		5	99999	5	5	99999
99999	-	-	1				
4	2	84	9	3	7	1	3
0	1	3	12	0	2006420	2000620	18
2	22	0.409090909		-	-	7	-
99999	0		5	0	7	3	99999
99999	-	-	1				
4	2	85	10	6	11	1	5
0	1	6	5	1	2042021	2010721	22
2	22	0.454545455		-	-	5	-
99999	99999		1	99999	5	5	99999
99999	-	-	1				
4	2	86	10	5	8	1	5
0	1	5	6	0	2005022	2018422	17
2	22	0.454545455		-	-	5	-
99999	0		1	0	5	5	99999
99999	-	-	1				
4	2	87	12	5	10	1	5
0	1	5	10	0	2037523	2052423	20
2	22	0.545454545		-	-	5	-
99999	0		5	0	5	5	99999
99999	-	-	1				
4	2	88	11	12	18	1	3
1	1	12	3	1	2082924	2017624	24
2	22	0.5	-	-	7	-	99999
99999		5	99999	7	3	99999	
99999	-	-	1				
5	1	89	4	3	3	0	
7	-1	1	3	7	1	1069801	1023201
13	1	20	0.2	30	0	3	27
0	0		0	0	3	7	4
0	25	0	1				
5	1	90	8	4	4	0	
6	-1	1	4	6	1	1096602	1087902
16	1	20	0.4	29	0	4	
23	-	-		0	-	4	6
0	-	16	0	1			
5	1	91	3	4	4	0	
4	-1	1	4	6	0	1045803	1075003
8	1	20	0.15	29	34	6	
0	-	-		0	-	6	4
0	-	0	19	1			
5	1	92	2	5	5	0	
5	-1	1	5	5	1	1068304	1015004
12	1	20	0.1	30	0	5	
20	-	-		0	-	5	5
4	-	33	0	1			
5	1	93	7	3	3	0	
3	-1	1	3	7	0	1024805	1092805
2	1	20	0.35	30	43	7	
0	-	-		0	-	7	3
4	-	0	24	1			
5	1	94	5	8	8	0	
2	-1	1	8	2	1	1070706	1034306

14	1	20	0.25	30	0	8	
28	–	–		0	–	8	2
5	–	6	0	1			
5	1	95	3	6	6	0	
4	-1	1	6	4	1	1062607	1016207
11	1	20	0.15	29	0	6	
23	–	–		0	–	6	4
4	–	26	0	1			
5	1	96	1	4	4	0	
4	-1	1	4	6	0	1040708	1004708
5	1	20	0.05	30	99999	6	
0	–	–		0	–	6	4
6	–	0	27	1			
5	1	97	5	2	2	0	
2	-1	1	2	8	0	1039109	1091509
4	1	20	0.25	30	45	8	
0	–	–		0	–	8	2
8	–	0	20	1			
5	1	98	7	7	7	0	
3	-1	1	7	3	1	1046510	1057810
9	1	20	0.35	30	0	7	
15	–	–		0	–	7	3
4	–	18	0	1			
5	1	99	6	5	5	0	
5	-1	1	5	5	0	1042411	1092111
6	1	20	0.3	30	45	5	
0	–	–		0	–	5	5
5	–	0	22	1			
5	1	100	2	5	5	0	
5	-1	1	5	5	0	1038112	1013012
3	1	20	0.1	30	45	5	
0	–	–		0	–	5	5
9	–	0	27	1			
5	1	101	4	7	7	0	
7	-1	1	7	3	0	1006813	1086513
1	1	20	0.2	30	45	3	
0	–	–		0	–	3	7
4	–	0	11	1			
5	1	102	6	5	5	0	
5	-1	1	5	5	1	1081214	1056514
15	1	20	0.3	30	0	5	
20	–	–		0	–	5	5
4	–	22	0	1			
5	1	103	8	6	6	0	
6	-1	1	6	4	0	1045115	1093915
7	1	20	0.4	30	45	4	
0	–	–		0	–	4	6
0	–	0	20	1			
5	1	104	1	6	6	0	
4	-1	1	6	4	1	1051116	1006916
10	1	20	0.05	30	0	6	
15	–	–		0	–	6	4
4	–	22	0	1			
5	1	105	9	5	5	0	

5	-1	1	5	5	0	2004817	2012517
18	2	20	0.45	30	23	5	
0	-	-		0	-	5	5
4	-	0	19	1			
5	1	106	10	5	5	0	
5	-1	1	5	5	0	2004118	2037618
17	2	20	0.5	30	19	5	
0	-	-		0	-	5	5
4	-	0	20	1			
5	1	107	11	5	5	0	
5	-1	1	5	5	1	2066519	2082319
22	2	20	0.55	30	0	5	
27	-	-		0	-	5	5
6	-	45	0	1			
5	1	108	10	5	5	0	
5	-1	1	5	5	1	2030920	2061320
20	2	20	0.5	29	0	5	
27	-	-		0	-	5	5
5	-	37	0	1			
5	1	109	9	5	5	0	
5	-1	1	5	5	1	2045821	2048321
21	2	20	0.45	30	0	5	
27	-	-		0	-	5	5
4	-	25	0	1			
5	1	110	11	5	5	0	
5	-1	1	5	5	0	2019922	2071122
19	2	20	0.55	30	41	5	
0	-	-		0	-	5	5
0	-	0	21	1			
5	2	89	7	0	3	1	3
1	0	0	0	1	1069801	1077501	13
1	20	0.35	-	-	7	-	12
99999		0	99999	0	0	99999	
99999	-	-	1				
5	2	90	8	9	13	1	5
0	1	9	5	1	1096602	1081002	16
1	20	0.4	-	-	5	-	-20
99999		4	99999	5	5	99999	
99999	-	-	1				
5	2	91	4	5	9	1	5
0	1	5	10	0	1045803	1063603	8
1	20	0.2	-	-	5	-	32
0		5	0	5	5	99999	
99999	-	-	1				
5	2	92	4	10	15	1	5
0	1	10	5	1	1068304	1051404	12
1	20	0.2	-	-	5	-	-21
99999		5	99999	5	5	99999	
99999	-	-	1				
5	2	93	2	4	7	1	4
1	1	4	11	0	1024805	1025805	2
1	20	0.1	-	-	6	-	39
0		5	0	6	4	99999	
99999	-	-	1				

5	2	94	6	10	18	1	2
1	1	10	2	1	1070706	1063406	14
1	20	0.3	–	–	8	–	15
99999		2	99999	8	2	99999	
99999	–	–	1				
5	2	95	1	10	16	1	4
0	1	10	4	1	1062607	1002807	11
1	20	0.05	–	–	6	–	1
99999		4	99999	6	4	99999	
99999	–	–	1				
5	2	96	3	6	10	1	6
1	1	6	9	0	1040708	1037408	5
1	20	0.15	–	–	4	–	40
0		5	0	4	6	99999	
99999	–	–	1				
5	2	97	6	2	4	1	2
1	1	2	10	0	1039109	1076709	4
1	20	0.3	–	–	8	–	44
0		2	0	8	2	99999	
99999	–	–	1				
5	2	98	5	10	17	1	2
1	1	10	2	1	1046510	1053310	9
1	20	0.25	–	–	8	–	3
99999		2	99999	8	2	99999	
99999	–	–	1				
5	2	99	5	2	7	1	2
1	1	2	10	0	1042411	1068911	6
1	20	0.25	–	–	8	–	39
0		2	0	8	2	99999	
99999	–	–	1				
5	2	100	1	4	9	1	4
0	1	4	10	0	1038112	1013612	3
1	20	0.05	–	–	6	–	39
0		4	0	6	4	99999	
99999	–	–	1				
5	2	101	7	0	7	1	3
1	0	0	0	0	1006813	1082713	1
1	20	0.35	–	–	7	–	38
0		0	0	0	0	99999	
99999	–	–	1				
5	2	102	3	9	14	1	6
1	1	9	6	1	1081214	1044314	15
1	20	0.15	–	–	4	–	-8
99999		5	99999	4	6	99999	
99999	–	–	1				
5	2	103	8	5	11	1	5
0	1	5	9	0	1045115	1086615	7
1	20	0.4	–	–	5	–	40
0		4	0	5	5	99999	
99999	–	–	1				
5	2	104	2	11	17	1	4
1	1	11	4	1	1051116	1025716	10
1	20	0.1	–	–	6	–	-6
99999		5	99999	6	4	99999	

99999	-	-	1				
5	2	105	11	0	5	1	7
1	0	0	0	0	2004817	2075917	18
2	20	0.55	-	-	3	-	40
0		0	0	0	0	99999	
99999	-	-	1				
5	2	106	10	5	10	1	5
0	1	5	10	0	2004118	2038918	17
2	20	0.5	-	-	5	-	35
0		5	0	5	5	99999	
99999	-	-	1				
5	2	107	10	10	15	1	5
0	1	10	5	1	2066519	2038219	22
2	20	0.5	-	-	5	-	14
99999		5	99999	5	5	99999	
99999	-	-	1				
5	2	108	9	8	13	1	4
0	1	8	4	1	2030920	2038120	20
2	20	0.45	-	-	6	-	19
99999		2	99999	6	4	99999	
99999	-	-	1				
5	2	109	11	0	5	1	7
1	0	0	0	1	2045821	2056821	21
2	20	0.55	-	-	3	-	-35
99999		0	99999	0	0	99999	
99999	-	-	1				
5	2	110	9	4	9	1	4
0	1	4	8	0	2019922	2022922	19
2	20	0.45	-	-	6	-	34
0		2	0	6	4	99999	
99999	-	-	1				
6	1	111	7	4	4	0	
4	-1	1	4	6	0	1045801	1055301
8	1	20	0.35	30	43	6	0
0	0		0	0	6	4	99999
0	0	99999	1				
6	1	112	6	5	5	0	
5	-1	1	5	5	0	1034102	1050302
6	1	20	0.3	30	99999	5	
0	-	-		0	-	5	5
99999	-	0	99999	1			
6	1	113	6	5	5	0	
5	-1	1	5	5	1	1058903	1048603
10	1	20	0.3	30	0	5	
99999	-	-		0	-	5	5
99999	-	99999	0	1			
6	1	114	4	5	5	0	
5	-1	1	5	5	1	1093804	1021304
15	1	20	0.2	30	0	5	
99999	-	-		0	-	5	5
99999	-	36	0	1			
6	1	115	2	6	6	0	
4	-1	1	6	4	1	1078705	1014905
13	1	20	0.1	30	0	6	

99999	–	–		0	–	6	4
99999	–	36	0	1			
6	1	116	2	4	4	0	
4	-1	1	4	6	0	1017706	1003106
3	1	20	0.1	30	99999	6	
0	–	–		0	–	6	4
99999	–	0	99999	1			
6	1	117	8	6	6	0	
4	-1	1	6	4	1	1071907	1074007
12	1	20	0.4	30	0	6	
99999	–	–		0	–	6	4
99999	–	36	0	1			
6	1	118	7	6	6	0	
4	-1	1	6	4	1	1047708	1072208
9	1	20	0.35	30	0	6	
99999	–	–		0	–	6	4
99999	–	36	0	1			
6	1	119	8	4	4	0	
4	-1	1	4	6	0	1031309	1066309
5	1	20	0.4	30	36	6	
0	–	–		0	–	6	4
99999	–	0	99999	1			
6	1	120	1	5	5	0	
5	-1	1	5	5	0	1007110	1002710
2	1	20	0.05	30	40	5	
0	–	–		0	–	5	5
99999	–	0	99999	1			
6	1	121	3	4	4	0	
4	-1	1	4	6	0	1006311	1016711
1	1	20	0.15	30	36	6	
0	–	–		0	–	6	4
99999	–	0	99999	1			
6	1	122	1	5	5	0	
5	-1	1	5	5	1	1087712	1000812
14	1	20	0.05	30	0	5	
99999	–	–		0	–	5	5
99999	–	36	0	1			
6	1	123	5	0	0	0	
3	-1	0	0	0	0	1030413	1022913
4	1	20	0.25	30	44	7	
0	–	–		0	–	0	0
99999	–	0	99999	1			
6	1	124	4	5	5	0	
5	-1	1	5	5	0	1042314	1020814
7	1	20	0.2	30	39	5	
0	–	–		0	–	5	5
99999	–	0	99999	1			
6	1	125	3	6	6	0	
4	-1	1	6	4	1	1060415	1018815
11	1	20	0.15	30	0	6	
99999	–	–		0	–	6	4
99999	–	36	0	1			
6	1	126	5	0	0	0	
3	-1	0	0	0	1	1099716	1047016

16	1	20	0.25	30	0	7	
99999	-	-		0	-	0	0
99999	-	36	0	1			
6	1	127	10	5	5	0	
5	-1	1	5	5	1	2047617	2023117
21	2	20	0.5	30	0	5	
99999	-	-		0	-	5	5
99999	-	38	0	1			
6	1	128	10	5	5	0	
5	-1	1	5	5	0	2007718	2082418
18	2	20	0.5	30	44	5	
0	-	-		0	-	5	5
99999	-	0	99999	1			
6	1	129	9	6	6	0	
4	-1	1	6	4	1	2071419	2002019
22	2	20	0.45	30	0	6	
99999	-	-		0	-	6	4
99999	-	36	0	1			
6	1	130	9	4	4	0	
4	-1	1	4	6	0	2004220	2014520
17	2	20	0.45	30	36	6	
0	-	-		0	-	6	4
99999	-	0	99999	1			
6	1	131	11	0	0	0	
3	-1	0	0	0	1	2014421	2037421
20	2	20	0.55	30	0	7	
99999	-	-		0	-	0	0
99999	-	36	0	1			
6	1	132	11	0	0	0	
3	-1	0	0	0	0	2008822	2097222
19	2	20	0.55	27	42	7	
0	-	-		0	-	0	0
99999	-	0	99999	1			
6	2	111	1	0	4	1	3
1	0	0	0	0	1045801	1014101	8
1	20	0.05	-	-	7	-	99999
0		0	0	0	0	99999	
99999	-	-	1				
6	2	112	8	6	11	1	6
0	1	6	6	0	1034102	1090402	6
1	20	0.4	-	-	4	-	99999
0		2	0	4	6	99999	
99999	-	-	1				
6	2	113	3	10	15	1	5
1	1	10	5	1	1058903	1037903	10
1	20	0.15	-	-	5	-	99999
99999		5	99999	5	5	99999	
99999	-	-	1				
6	2	114	4	12	17	1	3
0	1	12	3	1	1093804	1065804	15
1	20	0.2	-	-	7	-	99999
99999		5	99999	7	3	99999	
99999	-	-	1				
6	2	115	1	0	6	1	3

1	0	0	0	1	1078705	1012605	13
1	20	0.05	–	–	7	–	99999
99999		0	99999	0	0	99999	
99999	–	–	1				
6	2	116	3	5	9	1	5
1	1	5	10	0	1017706	1020306	3
1	20	0.15	–	–	5	–	99999
0		5	0	5	5	99999	
99999	–	–	1				
6	2	117	5	12	18	1	3
0	1	12	3	1	1071907	1066807	12
1	20	0.25	–	–	7	–	99999
99999		5	99999	7	3	99999	
99999	–	–	1				
6	2	118	7	11	17	1	4
0	1	11	4	1	1047708	1081208	9
1	20	0.35	–	–	6	–	99999
99999		5	99999	6	4	99999	
99999	–	–	1				
6	2	119	7	4	8	1	4
0	1	4	11	0	1031309	1085109	5
1	20	0.35	–	–	6	–	99999
0		5	0	6	4	99999	
99999	–	–	1				
6	2	120	5	3	8	1	3
0	1	3	12	0	1007110	1052010	2
1	20	0.25	–	–	7	–	99999
0		5	0	7	3	99999	
99999	–	–	1				
6	2	121	4	3	7	1	3
0	1	3	12	0	1006311	1034311	1
1	20	0.2	–	–	7	–	99999
0		5	0	7	3	99999	
99999	–	–	1				
6	2	122	8	6	11	1	6
0	1	6	6	1	1087712	1088612	14
1	20	0.4	–	–	4	–	99999
99999		2	99999	4	6	99999	
99999	–	–	1				
6	2	123	6	0	0	1	3
1	0	0	0	0	1030413	1078013	4
1	20	0.3	–	–	7	–	99999
0		0	0	0	0	99999	
99999	–	–	1				
6	2	124	2	5	10	1	5
1	1	5	10	0	1042314	1019414	7
1	20	0.1	–	–	5	–	99999
0		5	0	5	5	99999	
99999	–	–	1				
6	2	125	6	0	6	1	3
1	0	0	0	1	1060415	1073115	11
1	20	0.3	–	–	7	–	99999
99999		0	99999	0	0	99999	
99999	–	–	1				

6	2	126	2	10	10	1	5
1	1	10	5	1	1099716	1028316	16
1	20	0.1	–	–	5	–	99999
99999		5	99999	5	5	99999	
99999	–	–	1				
6	2	127	11	10	15	1	5
0	1	10	5	1	2047617	2030817	21
2	20	0.55	–	–	5	–	99999
99999		5	99999	5	5	99999	
99999	–	–	1				
6	2	128	10	4	9	1	4
1	1	4	9	0	2007718	2052618	18
2	20	0.5	–	–	6	–	99999
0		3	0	6	4	99999	
99999	–	–	1				
6	2	129	10	9	15	1	4
1	1	9	4	1	2071419	2022219	22
2	20	0.5	–	–	6	–	99999
99999		3	99999	6	4	99999	
99999	–	–	1				
6	2	130	9	0	4	1	3
1	0	0	3	0	2004220	2029020	17
2	20	0.45	–	–	7	–	99999
0		3	0	0	0	99999	
99999	–	–	1				
6	2	131	9	3	3	1	3
1	0	3	0	1	2014421	2016021	20
2	20	0.45	–	–	7	–	99999
99999		3	99999	0	0	99999	
99999	–	–	1				
6	2	132	11	5	5	1	5
0	1	5	10	0	2008822	2069822	19
2	20	0.55	–	–	5	–	99999
0		5	0	5	5	99999	
99999	–	–	1				
7	1	133	2	5	5	0	
5	-1	1	5	5	0	1025901	1042901
5	1	16	0.125	30	44	5	0
0	0	0	0	0	5	5	99999
0	0	99999	2				
7	1	134	5	4	4	0	
4	-1	1	4	6	0	1023202	1076302
4	1	16	0.3125	30	99999	6	
0	–	–	–	0	–	6	4
99999	–	0	99999	2			
7	1	135	6	0	0	0	
2	-1	0	0	0	1	1076803	1059603
13	1	16	0.375	30	0	8	
99999	–	–	–	0	–	0	0
99999	–	22	0	2			
7	1	136	7	0	0	0	
3	-1	0	0	0	0	1013304	1093304
1	1	16	0.4375	25	14	7	
0	–	–	–	0	–	0	0

99999	–	0	99999	2			
7	1	137	4	0	0	0	
2	-1	0	0	0	1	1075005	1042905
12	1	16	0.25	30	0	8	
99999	–	–	–	0	–	0	0
99999	–	30	0	2			
7	1	138	8	5	5	0	
5	-1	1	5	5	1	1073506	1061606
11	1	16	0.5	30	0	5	
99999	–	–	–	0	–	5	5
99999	–	38	0	2			
7	1	139	1	5	5	0	
5	-1	1	5	5	0	1044407	1006307
8	1	16	0.0625	30	29	5	
0	–	–	–	0	–	5	5
99999	–	0	99999	2			
7	1	140	6	0	0	0	
2	-1	0	0	0	0	1027808	1076908
6	1	16	0.375	30	99999	8	
0	–	–	–	0	–	0	0
99999	–	0	99999	2			
7	1	141	3	5	5	0	
5	-1	1	5	5	0	1013309	1066509
2	1	16	0.1875	30	27	5	
0	–	–	–	0	–	5	5
99999	–	0	99999	2			
7	1	142	4	0	0	0	
2	-1	0	0	0	0	1036310	1068610
7	1	16	0.25	30	41	8	
0	–	–	–	0	–	0	0
99999	–	0	99999	2			
7	1	143	3	5	5	0	
5	-1	1	5	5	1	1068111	1038011
10	1	16	0.1875	30	0	5	
99999	–	–	–	0	–	5	5
99999	–	7	0	2			
7	1	144	1	5	5	0	
5	-1	1	5	5	1	1084312	1014612
14	1	16	0.0625	30	0	5	
99999	–	–	–	0	–	5	5
99999	–	36	0	2			
7	1	145	7	0	0	0	
3	-1	0	0	0	1	1098513	1060913
16	1	16	0.4375	30	0	7	
99999	–	–	–	0	–	0	0
99999	–	15	0	2			
7	1	146	8	5	5	0	
5	-1	1	5	5	0	1015714	1098314
3	1	16	0.5	30	99999	5	
0	–	–	–	0	–	5	5
99999	–	0	99999	2			
7	1	147	2	5	5	0	
5	-1	1	5	5	1	1055215	1036315
9	1	16	0.125	30	0	5	

99999	–	–	–	0	–	5	5
99999	–	25	0	2			
7	1	148	5	6	6	0	
4	-1	1	6	4	1	1094416	1048516
15	1	16	0.3125	30	0	6	
99999	–	–	–	0	–	6	4
99999	–	27	0	2			
7	1	149	9	4	4	0	
6	-1	1	4	6	1	2081417	2052817
18	2	16	0.5625	25	0	4	
99999	–	–	–	0	–	4	6
99999	–	12	0	2			
7	1	150	9	6	6	0	
6	-1	1	6	4	0	2028718	2003318
17	2	16	0.5625	30	42	4	
0	–	–	–	0	–	4	6
99999	–	0	99999	2			
7	2	133	4	5	10	1	5
0	1	5	10	0	1025901	1045301	5
1	16	0.25	–	–	5	–	99999
99999	0	5	0	5	5	99999	
99999	–	–	2				
7	2	134	2	0	4	1	8
0	0	0	1	0	1023202	1020802	4
1	16	0.125	–	–	2	–	99999
99999	0	1	0	0	0	99999	
99999	–	–	2				
7	2	135	3	0	0	1	2
1	0	0	0	1	1076803	1040603	13
1	16	0.1875	–	–	8	–	0
0	99999	0	99999	0	0	99999	
99999	–	–	2				
7	2	136	6	0	0	1	4
0	0	0	0	0	1013304	1070404	1
1	16	0.375	–	–	6	–	99999
99999	0	0	0	0	0	99999	
99999	–	–	2				
7	2	137	5	7	7	1	3
1	1	7	3	1	1075005	1050205	12
1	16	0.3125	–	–	7	–	0
0	99999	0	99999	7	3	99999	
99999	–	–	2				
7	2	138	4	10	15	1	5
0	1	10	5	1	1073506	1041806	11
1	16	0.25	–	–	5	–	0
0	99999	5	99999	5	5	99999	
99999	–	–	2				
7	2	139	7	5	10	1	5
0	1	5	10	0	1044407	1082307	8
1	16	0.4375	–	–	5	–	99999
99999	0	5	0	5	5	99999	
99999	–	–	2				
7	2	140	3	0	0	1	2
1	0	0	0	0	1027808	1041108	6

1	16	0.1875	–	–	8	–	99999
99999	0	0	0	0	0	99999	
99999	–	–	2				
7	2	141	1	7	12	1	7
1	1	7	4	0	1013309	1002109	2
1	16	0.0625	–	–	3	–	99999
99999	0	1	0	3	7	99999	
99999	–	–	2				
7	2	142	8	1	1	1	1
0	1	1	9	0	1036310	1086210	7
1	16	0.5	–	–	9	–	99999
99999	0	0	0	9	1	99999	
99999	–	–	2				
7	2	143	2	1	6	1	8
0	0	1	0	1	1068111	1028311	10
1	16	0.125	–	–	2	–	0
0	99999	1	99999	0	0	99999	
99999	–	–	2				
7	2	144	7	10	15	1	5
0	1	10	5	1	1084312	1068612	14
1	16	0.4375	–	–	5	–	0
0	99999	5	99999	5	5	99999	
99999	–	–	2				
7	2	145	8	9	9	1	1
0	1	9	1	1	1098513	1082013	16
1	16	0.5	–	–	9	–	0
0	99999	0	99999	9	1	99999	
99999	–	–	2				
7	2	146	5	3	8	1	3
1	1	3	7	0	1015714	1063014	3
1	16	0.3125	–	–	7	–	99999
99999	0	0	0	7	3	99999	
99999	–	–	2				
7	2	147	1	4	9	1	7
1	1	4	7	1	1055215	1009915	9
1	16	0.0625	–	–	3	–	0
0	99999	1	99999	3	7	99999	
99999	–	–	2				
7	2	148	6	0	6	1	4
0	0	0	0	1	1094416	1051116	15
1	16	0.375	–	–	6	–	0
0	99999	0	99999	0	0	99999	
99999	–	–	2				
7	2	149	9	0	4	1	2
0	0	0	0	1	2081417	2055517	18
2	16	0.5625	–	–	8	–	0
0	99999	0	99999	0	0	99999	
99999	–	–	2				
7	2	150	9	0	6	1	2
0	0	0	0	0	2028718	2000418	17
2	16	0.5625	–	–	8	–	99999
99999	0	0	0	0	0	99999	
99999	–	–	2				
8	1	151	4	0	0	0	

4	-1	0	0	0	1	1072001	1054201
12	1	22	0.181818182		30	0	6
99999	0	0	0	0	0	0	0
99999	0	32	0	2			
8	1	152	3	2	2	0	
8	-1	1	2	8	1	1099102	1044502
16	1	22	0.136363636		30	0	2
99999	-	-	-	0	-	2	8
99999	-	32	0	2			
8	1	153	3	8	8	0	
8	-1	1	8	2	0	1030703	1044003
4	1	22	0.136363636		30	99999	2
0	-	-	-	0	-	2	8
99999	-	0	99999	2			
8	1	154	2	5	5	0	
5	-1	1	5	5	1	1079404	1033504
14	1	22	0.090909091		30	0	5
99999	-	-	-	0	-	5	5
99999	-	32	0	2			
8	1	155	1	5	5	0	
5	-1	1	5	5	1	1072605	1009405
13	1	22	0.045454545		30	0	5
99999	-	-	-	0	-	5	5
99999	-	32	0	2			
8	1	156	5	4	4	0	
4	-1	1	4	6	0	1042906	1045406
6	1	22	0.227272727		30	45	6
0	-	-	-	0	-	6	4
99999	-	0	99999	2			
8	1	157	4	0	0	0	
4	-1	0	0	0	0	1030307	1044707
3	1	22	0.181818182		30	43	6
0	-	-	-	0	-	0	0
99999	-	0	99999	2			
8	1	158	6	5	5	0	
5	-1	1	5	5	0	1054508	1045608
7	1	22	0.272727273		30	43	5
0	-	-	-	0	-	5	5
99999	-	0	99999	2			
8	1	159	8	5	5	0	
5	-1	1	5	5	1	1064209	1075509
10	1	22	0.363636364		30	0	5
99999	-	-	-	0	-	5	5
99999	-	36	0	2			
8	1	160	7	4	4	0	
6	-1	1	4	6	1	1062810	1073610
9	1	22	0.318181818		30	0	4
99999	-	-	-	0	-	4	6
99999	-	32	0	2			
8	1	161	8	5	5	0	
5	-1	1	5	5	0	1040111	1067711
5	1	22	0.363636364		30	99999	5
0	-	-	-	0	-	5	5
99999	-	0	99999	2			

8	1	162	2	5	5	0	
5	-1	1	5	5	0	1062012	1040712
8	1	22	0.090909091		30	32	5
0	-	-	-	0	-	5	5
99999	-	0	99999	2			
8	1	163	7	6	6	0	
6	-1	1	6	4	0	1015213	1053313
2	1	22	0.318181818		30	43	4
0	-	-	-	0	-	4	6
99999	-	0	99999	2			
8	1	164	5	6	6	0	
4	-1	1	6	4	1	1092114	1056614
15	1	22	0.227272727		23	0	6
99999	-	-	-	0	-	6	4
99999	-	39	0	2			
8	1	165	6	5	5	0	
5	-1	1	5	5	1	1071715	1073115
11	1	22	0.272727273		30	0	5
99999	-	-	-	0	-	5	5
99999	-	32	0	2			
8	1	166	1	5	5	0	
5	-1	1	5	5	0	1010516	1022916
1	1	22	0.045454545		30	42	5
0	-	-	-	0	-	5	5
99999	-	0	99999	2			
8	1	167	9	0	0	0	
4	-1	0	0	0	0	2019817	2008617
19	2	22	0.409090909		30	41	6
0	-	-	-	0	-	0	0
99999	-	0	99999	2			
8	1	168	12	0	0	0	
4	-1	0	0	0	1	2065118	2050318
22	2	22	0.545454545		30	0	6
99999	-	-	-	0	-	0	0
99999	-	32	0	2			
8	1	169	11	3	3	0	
3	-1	1	3	7	0	2029619	2048819
20	2	22	0.5	30	43	7	
0	-	-	-	0	-	7	3
99999	-	0	99999	2			
8	1	170	11	7	7	0	
3	-1	1	7	3	1	2096420	2048020
24	2	22	0.5	30	0	7	
99999	-	-	-	0	-	7	3
99999	-	32	0	2			
8	1	171	9	0	0	0	
4	-1	0	0	0	1	2088121	2001121
23	2	22	0.409090909		30	0	6
99999	-	-	-	0	-	0	0
99999	-	32	0	2			
8	1	172	10	0	0	0	
0	-1	0	0	0	1	2054522	2015122
21	2	22	0.454545455		30	0	10
99999	-	-	-	0	-	0	0

99999	–	32	0	2			
8	1	173	12	0	0	0	
4	-1	0	0	0	0	2019323	2051423
17	2	22	0.545454545		30	42	6
0	–	–	–	0	–	0	0
99999	–	0	99999	2			
8	1	174	10	0	0	0	
0	-1	0	0	0	0	2019524	2023324
18	2	22	0.454545455		27	35	10
0	–	–	–	0	–	0	0
99999	–	0	99999	2			
8	2	151	6	6	6	1	4
0	1	6	4	1	1072001	1067601	12
1	22	0.272727273		–	–	6	–
0	0	99999	0	99999	6	4	99999
99999	–	–	2				
8	2	152	1	13	15	1	2
1	1	13	2	1	1099102	1007402	16
1	22	0.045454545		–	–	8	–
0	0	99999	5	99999	8	2	99999
99999	–	–	2				
8	2	153	2	5	13	1	5
0	1	5	9	0	1030703	1016903	4
1	22	0.090909091		–	–	5	–
99999	99999	0	4	0	5	5	99999
99999	–	–	2				
8	2	154	8	5	10	1	5
0	1	5	5	1	1079404	1080504	14
1	22	0.363636364		–	–	5	–
0	0	99999	0	99999	5	5	99999
99999	–	–	2				
8	2	155	7	9	14	1	6
1	1	9	6	1	1072605	1076505	13
1	22	0.318181818		–	–	4	–
0	0	99999	5	99999	4	6	99999
99999	–	–	2				
8	2	156	4	4	8	1	4
1	1	4	6	0	1042906	1040906	6
1	22	0.181818182		–	–	6	–
99999	99999	0	0	0	6	4	99999
99999	–	–	2				
8	2	157	8	5	5	1	5
0	1	5	5	0	1030307	1099007	3
1	22	0.363636364		–	–	5	–
99999	99999	0	0	0	5	5	99999
99999	–	–	2				
8	2	158	5	5	10	1	5
0	1	5	10	0	1054508	1048108	7
1	22	0.227272727		–	–	5	–
99999	99999	0	5	0	5	5	99999
99999	–	–	2				
8	2	159	3	6	11	1	4
1	1	6	4	1	1064209	1032609	10
1	22	0.136363636		–	–	6	–

0	0	99999	0	99999	6	4	99999
99999	-	-	2				
8	2	160	5	10	14	1	5
0	1	10	5	1	1062810	1052810	9
1	22	0.227272727		-	-	5	-
0	0	99999	5	99999	5	5	99999
99999	-	-	2				
8	2	161	7	6	11	1	6
1	1	6	9	0	1040111	1098011	5
1	22	0.318181818		-	-	4	-
99999	99999	0	5	0	4	6	99999
99999	-	-	2				
8	2	162	6	4	9	1	4
0	1	4	6	0	1062012	1092812	8
1	22	0.272727273		-	-	6	-
99999	99999	0	0	0	6	4	99999
99999	-	-	2				
8	2	163	1	2	8	1	2
1	1	2	13	0	1015213	1007113	2
1	22	0.045454545		-	-	8	-
99999	99999	0	5	0	8	2	99999
99999	-	-	2				
8	2	164	4	6	12	1	4
1	1	6	4	1	1092114	1039514	15
1	22	0.181818182		-	-	6	-
0	0	99999	0	99999	6	4	99999
99999	-	-	2				
8	2	165	2	9	14	1	5
0	1	9	5	1	1071715	1010015	11
1	22	0.090909091		-	-	5	-
0	0	99999	4	99999	5	5	99999
99999	-	-	2				
8	2	166	3	4	9	1	4
1	1	4	6	0	1010516	1033416	1
1	22	0.136363636		-	-	6	-
99999	99999	0	0	0	6	4	99999
99999	-	-	2				
8	2	167	10	4	4	1	4
0	1	4	7	0	2019817	2013917	19
2	22	0.454545455		-	-	6	-
99999	99999	0	1	0	6	4	99999
99999	-	-	2				
8	2	168	11	12	12	1	3
1	1	12	3	1	2065118	2046018	22
2	22	0.5	-	-	7	-	0
0	99999	5	99999	7	3	99999	
99999	-	-	2				
8	2	169	11	3	6	1	3
1	1	3	12	0	2029619	2017519	20
2	22	0.5	-	-	7	-	99999
99999	0	5	0	7	3	99999	
99999	-	-	2				
8	2	170	10	7	14	1	4
0	1	7	4	1	2096420	2019920	24

2	22	0.454545455		–	–	6	–
0	0	99999	1	99999	6	4	99999
99999	–	–	2				
8	2	171	9	6	6	1	4
0	1	6	4	1	2088121	2013821	23
2	22	0.409090909		–	–	6	–
0	0	99999	0	99999	6	4	99999
99999	–	–	2				
8	2	172	12	0	0	1	0
0	0	0	0	1	2054522	2055122	21
2	22	0.545454545		–	–	10	–
0	0	99999	0	99999	0	0	99999
99999	–	–	2				
8	2	173	12	0	0	1	0
0	0	0	0	0	2019323	2073523	17
2	22	0.545454545		–	–	10	–
99999	99999	0	0	0	0	0	99999
99999	–	–	2				
8	2	174	9	4	4	1	4
0	1	4	6	0	2019524	2010024	18
2	22	0.409090909		–	–	6	–
99999	99999	0	0	0	6	4	99999
99999	–	–	2				
9	1	175	4	5	5	0	
5	-1	1	5	5	1	1071301	1059201
10	1	12	0.333333333		29	0	5
99999	0	0	0	0	0	5	5
99999	0	42	0	2			
9	1	176	5	5	5	0	
5	-1	1	5	5	0	1053202	1039102
5	1	12	0.416666667		29	36	5
0	–	–	–	0	–	5	5
99999	–	0	99999	2			
9	1	177	5	5	5	0	
5	-1	1	5	5	1	1070603	1065603
9	1	12	0.416666667		29	0	5
99999	–	–	–	0	–	5	5
99999	–	10	0	2			
9	1	178	6	3	3	0	
3	-1	1	3	7	0	1030904	1057204
3	1	12	0.5	29	40	7	
0	–	–	–	0	–	7	3
99999	–	0	99999	2			
9	1	179	7	0	0	0	
2	-1	0	0	0	0	1016705	1078405
2	1	12	0.583333333		28	37	8
0	–	–	–	0	–	0	0
99999	–	0	99999	2			
9	1	180	3	4	4	0	
4	-1	1	4	6	0	1053206	1013506
6	1	12	0.25	29	7	6	
0	–	–	–	0	–	6	4
99999	–	0	99999	2			
9	1	181	1	7	7	0	

3	-1	1	7	3	1	1094607	1003207
13	1	12	0.083333333		29	0	7
99999	-	-	-	0	-	7	3
99999	-	38	0	2			
9	1	182	4	5	5	0	
5	-1	1	5	5	0	1010608	1023708
1	1	12	0.333333333		29	39	5
0	-	-	-	0	-	5	5
99999	-	0	99999	2			
9	1	183	7	0	0	0	
2	-1	0	0	0	1	1072609	1089509
11	1	12	0.583333333		29	0	8
99999	-	-	-	0	-	0	0
99999	-	18	0	2			
9	1	184	6	7	7	0	
3	-1	1	7	3	1	1096710	1075010
14	1	12	0.5	29	0	7	
99999	-	-	-	0	-	7	3
99999	-	20	0	2			
9	1	185	3	6	6	0	
4	-1	1	6	4	1	1061111	1051811
8	1	12	0.25	29	0	6	
99999	-	-	-	0	-	6	4
99999	-	16	0	2			
9	1	186	2	0	0	0	
2	-1	0	0	0	0	1032812	1009712
4	1	12	0.166666667		29	37	8
0	-	-	-	0	-	0	0
99999	-	0	99999	2			
9	1	187	1	3	3	0	
3	-1	1	3	7	0	1056313	1002113
7	1	12	0.083333333		29	43	7
0	-	-	-	0	-	7	3
99999	-	0	99999	2			
9	1	188	2	0	0	0	
2	-1	0	0	0	1	1094314	1040714
12	1	12	0.166666667		29	0	8
99999	-	-	-	0	-	0	0
99999	-	36	0	2			
9	2	175	5	6	11	1	6
1	1	6	6	1	1071301	1039701	10
1	12	0.416666667		-	-	4	-
0	0	99999	2	99999	4	6	99999
99999	-	-	2				
9	2	176	2	0	5	1	2
1	0	0	2	0	1053202	1027202	5
1	12	0.166666667		-	-	8	-
99999	99999	0	2	0	0	0	99999
99999	-	-	2				
9	2	177	1	10	15	1	3
1	1	10	3	1	1070603	1013403	9
1	12	0.083333333		-	-	7	-
0	0	99999	3	99999	7	3	99999
99999	-	-	2				

9	2	178	4	3	6	1	3
0	1	3	7	0	1030904	1063404	3
1	12	0.333333333		-	-	7	-
99999	99999	0	0	0	7	3	99999
99999	-	-	2				
9	2	179	7	4	4	1	4
1	1	4	6	0	1016705	1097505	2
1	12	0.583333333		-	-	6	-
99999	99999	0	0	0	6	4	99999
99999	-	-	2				
9	2	180	3	0	4	1	2
1	0	0	5	0	1053206	1043306	6
1	12	0.25	-	-	8	-	99999
99999	0	5	0	0	0	99999	
99999	-	-	2				
9	2	181	4	7	14	1	3
0	1	7	3	1	1094607	1039207	13
1	12	0.333333333		-	-	7	-
0	0	99999	0	99999	7	3	99999
99999	-	-	2				
9	2	182	1	3	8	1	3
1	1	3	10	0	1010608	1017708	1
1	12	0.083333333		-	-	7	-
99999	99999	0	3	0	7	3	99999
99999	-	-	2				
9	2	183	3	5	5	1	2
1	0	5	0	1	1072609	1023709	11
1	12	0.25	-	-	8	-	0
0	99999	5	99999	0	0	99999	
99999	-	-	2				
9	2	184	2	2	9	1	2
1	0	2	0	1	1096710	1020510	14
1	12	0.166666667		-	-	8	-
0	0	99999	2	99999	0	0	99999
99999	-	-	2				
9	2	185	7	6	12	1	4
1	1	6	4	1	1061111	1085311	8
1	12	0.583333333		-	-	6	-
0	0	99999	0	99999	6	4	99999
99999	-	-	2				
9	2	186	6	2	2	1	2
0	1	2	9	0	1032812	1089612	4
1	12	0.5	-	-	8	-	99999
99999	0	1	0	8	2	99999	
99999	-	-	2				
9	2	187	5	6	9	1	6
1	1	6	6	0	1056313	1070813	7
1	12	0.416666667		-	-	4	-
99999	99999	0	2	0	4	6	99999
99999	-	-	2				
9	2	188	6	9	9	1	2
0	1	9	2	1	1094314	1051714	12
1	12	0.5	-	-	8	-	0
0	99999	1	99999	8	2	99999	

99999	-	-	2				
10	1	189	1	5	5	0	
5	-1	1	5	5	1	1059701	1033101
7	1	8	0.125	28	0	5	23
0	0	0	0	0	5	5	6
0	40	0	2				
10	1	190	4	0	0	0	
2	-1	0	0	0	0	1045102	1090102
3	1	8	0.5	28	40	8	
0	-	-	-	0	-	0	0
3	-	0	19	2			
10	1	191	4	0	0	0	
2	-1	0	0	0	1	1079403	1075403
9	1	8	0.5	28	0	8	
26	-	-	-	0	-	0	0
4	-	23	0	2			
10	1	192	2	6	6	0	
4	-1	1	6	4	1	1059504	1062204
6	1	8	0.25	27	0	6	
22	-	-	-	0	-	6	4
0	-	32	0	2			
10	1	193	3	7	7	0	
7	-1	1	7	3	0	1030505	1089605
2	1	8	0.375	28	41	3	
0	-	-	-	0	-	3	7
0	-	0	23	2			
10	1	194	3	3	3	0	
7	-1	1	3	7	1	1064306	1066406
8	1	8	0.375	28	0	3	
24	-	-	-	0	-	3	7
5	-	29	0	2			
10	1	195	5	5	5	0	
5	-1	1	5	5	1	1081207	1097007
10	1	8	0.625	28	0	5	
28	-	-	-	0	-	5	5
6	-	42	0	2			
10	1	196	2	4	4	0	
4	-1	1	4	6	0	1048108	1088508
4	1	8	0.25	28	41	6	
0	-	-	-	0	-	6	4
0	-	0	14	2			
10	1	197	5	5	5	0	
5	-1	1	5	5	0	1010409	1097809
1	1	8	0.625	22	42	5	
0	-	-	-	0	-	5	5
8	-	0	11	2			
10	1	198	1	5	5	0	
5	-1	1	5	5	0	1048410	1003210
5	1	8	0.125	28	41	5	
0	-	-	-	0	-	5	5
4	-	0	25	2			
10	2	189	2	6	11	1	5
0	1	6	5	1	1059701	1018001	7
1	8	0.25	-	-	5	-	0

0	99999	1	99999	5	5	99999	
99999	-	-	2				
10	2	190	2	5	5	1	5
0	1	5	6	0	1045102	1073002	3
1	8	0.25	-	-	5	-	-1
99999	0	1	0	5	5	99999	
99999	-	-	2				
10	2	191	5	10	10	1	3
1	1	10	3	1	1079403	1079803	9
1	8	0.625	-	-	7	-	0
0	99999	3	99999	7	3	99999	
99999	-	-	2				
10	2	192	4	5	11	1	1
0	0	5	0	1	1059504	1069604	6
1	8	0.5	-	-	9	-	0
0	99999	5	99999	0	0	99999	
99999	-	-	2				
10	2	193	5	3	10	1	3
1	1	3	10	0	1030505	1091005	2
1	8	0.625	-	-	7	-	39
6	0	3	0	7	3	99999	
99999	-	-	2				
10	2	194	3	8	11	1	3
0	1	8	3	1	1064306	1053406	8
1	8	0.375	-	-	7	-	0
0	99999	1	99999	7	3	99999	
99999	-	-	2				
10	2	195	1	10	15	1	5
0	1	10	5	1	1081207	1009007	10
1	8	0.125	-	-	5	-	0
0	99999	5	99999	5	5	99999	
99999	-	-	2				
10	2	196	3	3	7	1	3
0	1	3	8	0	1048108	1073708	4
1	8	0.375	-	-	7	-	38
45	0	1	0	7	3	99999	
99999	-	-	2				
10	2	197	4	0	5	1	1
0	0	0	5	0	1010409	1083209	1
1	8	0.5	-	-	9	-	44
45	0	5	0	0	0	99999	
99999	-	-	2				
10	2	198	1	5	10	1	5
0	1	5	10	0	1048410	1027710	5
1	8	0.125	-	-	5	-	14
31	0	5	0	5	5	99999	
99999	-	-	2				
11	1	1	9	5	5	0	
5	-1	1	5	5	0	1004301	1085301
2	1	18	0.5	30	43	5	0
0	0	0	0	0	5	5	99999
0	0		0				
11	1	2	1	4	4	0	
6	-1	1	4	6	1	1068302	1009302

15	1	18	0.055555556	30	0	4	
99999	-	-	-	0	-	4	6
99999	-	43		0			
11	1	3	7	3	3	0	
3	-1	1	3	7	0	1006703	1081903
3	1	18	0.388888889	30	43	7	
0	-	-	-	0	-	7	3
99999	-	0		0			
11	1	4	7	7	7	0	
3	-1	1	7	3	1	1094404	1068304
20	1	18	0.388888889	30	0	7	
99999	-	-	-	0	-	7	3
99999	-	43		0			
11	1	5	10	5	5	0	
5	-1	1	5	5	0	1028405	1098605
9	1	18	0.555555556	30	43	5	
0	-	-	-	0	-	5	5
99999	-	0		0			
11	1	6	2	6	6	0	
4	-1	1	6	4	1	1078406	1010106
19	1	18	0.111111111	30	0	6	
99999	-	-	-	0	-	6	4
99999	-	43		0			
11	1	7	3	5	5	0	
5	-1	1	5	5	1	1064207	1010607
14	1	18	0.166666667	30	0	5	
99999	-	-	-	0	-	5	5
99999	-	43		0			
11	1	8	8	5	5	0	
5	-1	1	5	5	1	1037008	1088408
12	1	18	0.444444444	30	0	5	
99999	-	-	-	0	-	5	5
99999	-	43		0			
11	1	9	6	7	7	0	
3	-1	1	7	3	1	1075809	1048009
17	1	18	0.333333333	27	0	7	
99999	-	-	-	0	-	7	3
99999	-	99999		0			
11	1	10	8	5	5	0	
5	-1	1	5	5	0	1016910	1083710
6	1	18	0.444444444	27	99999	5	
0	-	-	-	0	-	5	5
99999	-	0		0			
11	1	11	4	4	4	0	
4	-1	1	4	6	0	1008611	1034411
5	1	18	0.222222222	30	43	6	
0	-	-	-	0	-	6	4
99999	-	0		0			
11	1	12	10	5	5	0	
5	-1	1	5	5	1	1053612	1092912
13	1	18	0.555555556	30	0	5	
99999	-	-	-	0	-	5	5
99999	-	43		0			
11	1	13	5	6	6	0	

4	-1	1	6	4	1	1077513	1039913
18	1	18	0.277777778		30	0	6
99999	-	-	-	0	-	6	4
99999	-	43		0			
11	1	14	5	4	4	0	
4	-1	1	4	6	0	1032814	1047614
10	1	18	0.277777778		30	99999	6
0	-	-	-	0	-	6	4
99999	-	0		0			
11	1	15	2	4	4	0	
4	-1	1	4	6	0	1025015	1027115
7	1	18	0.111111111		30	43	6
0	-	-	-	0	-	6	4
99999	-	0		0			
11	1	16	3	5	5	0	
5	-1	1	5	5	0	1000616	1031116
1	1	18	0.166666667		30	99999	5
0	-	-	-	0	-	5	5
99999	-	0		0			
11	1	17	9	5	5	0	
5	-1	1	5	5	1	1069217	1092317
16	1	18	0.5	30	0	5	
99999	-	-	-	0	-	5	5
99999	-	43		0			
11	1	18	4	6	6	0	
4	-1	1	6	4	1	1033518	1021018
11	1	18	0.222222222		30	0	6
99999	-	-	-	0	-	6	4
99999	-	43		0			
11	1	19	1	6	6	0	
6	-1	1	6	4	0	1025819	1008519
8	1	18	0.055555556		27	99999	4
0	-	-	-	0	-	4	6
99999	-	0		0			
11	1	20	6	3	3	0	
3	-1	1	3	7	0	1006720	1080720
4	1	18	0.333333333		30	43	7
0	-	-	-	0	-	7	3
99999	-	0		0			
11	2	1	10	6	11	1	
6	-1	1	6	5	0	1004301	1097901
2	1	18	0.555555556		-	-	
4	-	99999	99999	0	1	0	4
6	99999	99999	-		0		
11	2	2	2	7	11	1	
3	-1	1	7	3	1	1068302	1023802
15	1	18	0.111111111		-	-	
7	-	99999	0	99999	0	99999	7
3	99999	99999	-		0		
11	2	3	4	7	10	1	
7	-1	1	7	3	0	1006703	1035603
3	1	18	0.222222222		-	-	
3	-	99999	99999	0	0	0	3
7	99999	99999	-		0		

11	2	4	5	7	14	1	
8	-1	1	7	8	1	1094404	1041904
20	1	18	0.277777778		-	-	
2	-	99999	0	99999	5	99999	2
8	99999	99999	-		0		
11	2	5	8	5	10	1	
5	-1	1	5	10	0	1028405	1057505
9	1	18	0.444444444		-	-	
5	-	99999	99999	0	5	0	5
5	99999	99999	-		0		
11	2	6	9	12	18	1	
3	-1	1	12	3	1	1078406	1063206
19	1	18	0.5	-	-	7	-
99999	0	99999	5	99999	7	3	99999
99999	-		0				
11	2	7	7	9	14	1	
6	-1	1	9	6	1	1064207	1056207
14	1	18	0.388888889		-	-	
4	-	99999	0	99999	5	99999	4
6	99999	99999	-		0		
11	2	8	8	10	15	1	
5	-1	1	10	5	1	1037008	1060708
12	1	18	0.444444444		-	-	
5	-	99999	0	99999	5	99999	5
5	99999	99999	-		0		
11	2	9	4	3	10	1	
7	-1	1	3	7	1	1075809	1040909
17	1	18	0.222222222		-	-	
3	-	99999	0	99999	0	99999	3
7	99999	99999	-		0		
11	2	10	2	3	8	1	
3	-1	1	3	7	0	1016910	1009710
6	1	18	0.111111111		-	-	
7	-	99999	99999	0	0	0	7
3	99999	99999	-		0		
11	2	11	1	4	8	1	
4	-1	1	4	10	0	1008611	1000511
5	1	18	0.055555556		-	-	
6	-	99999	99999	0	4	0	6
4	99999	99999	-		0		
11	2	12	6	11	16	1	
4	-1	1	11	4	1	1053612	1050412
13	1	18	0.333333333		-	-	
6	-	99999	0	99999	5	99999	6
4	99999	99999	-		0		
11	2	13	1	10	16	1	
4	-1	1	10	4	1	1077513	1014513
18	1	18	0.055555556		-	-	
6	-	99999	0	99999	4	99999	6
4	99999	99999	-		0		
11	2	14	6	4	8	1	
4	-1	1	4	11	0	1032814	1053014
10	1	18	0.333333333		-	-	
6	-	99999	99999	0	5	0	6

4	99999	99999	-		0		
11	2	15	7	6	10	1	
6	-1	1	6	9	0	1025015	1053015
7	1	18	0.388888889		-	-	
4	-	99999	99999	0	5	0	4
6	99999	99999	-		0		
11	2	16	9	3	8	1	
3	-1	1	3	12	0	1000616	1086516
1	1	18	0.5	-	-	7	-
99999	99999	0	5	0	7	3	99999
99999	-		0				
11	2	17	10	5	10	1	
6	-1	1	5	6	1	1069217	1063917
16	1	18	0.555555556		-	-	
4	-	99999	0	99999	1	99999	4
6	99999	99999	-		0		
11	2	18	3	11	17	1	
4	-1	1	11	4	1	1033518	1029618
11	1	18	0.166666667		-	-	
6	-	99999	0	99999	5	99999	6
4	99999	99999	-		0		
11	2	19	5	8	14	1	
8	-1	1	8	7	0	1025819	1037019
8	1	18	0.277777778		-	-	
2	-	99999	99999	0	5	0	2
8	99999	99999	-		0		
11	2	20	3	4	7	1	
4	-1	1	4	11	0	1006720	1019720
4	1	18	0.166666667		-	-	
6	-	99999	99999	0	5	0	6
4	99999	99999	-		0		
12	1	1	2	6	6	0	
4	-1	1	6	4	1	1048501	1005501
10	1	16	0.125	29	0	6	99999
0	0	0	0	0	6	4	99999
0	30	0	0				
12	1	2	9	7	7	0	
7	-1	1	7	3	0	1020302	1084202
5	1	16	0.5625	24	35	3	
0	-	-	-	0	-	3	7
99999	-	0	99999	0			
12	1	3	1	6	6	0	
4	-1	1	6	4	1	1082103	1004003
16	1	16	0.0625	29	0	6	
99999	-	-	-	0	-	6	4
99999	-	31	0	0			
12	1	4	8	5	5	0	
5	-1	1	5	5	0	1005204	1080304
1	1	16	0.5	29	27	5	
0	-	-	-	0	-	5	5
99999	-	0	99999	0			
12	1	5	3	0	0	0	
2	-1	0	0	0	0	1045005	1032205
7	1	16	0.1875	29	37	8	

0	–	–	–	0	–	0	0
99999	–	0	99999	0			
12	1	6	5	0	0	0	
2	-1	0	0	0	0	1010506	1051506
2	1	16	0.3125	29	40	8	
0	–	–	–	0	–	0	0
99999	–	0	99999	0			
12	1	7	6	4	4	0	
4	-1	1	4	6	0	1039607	1069307
6	1	16	0.375	29	40	6	
0	–	–	–	0	–	6	4
99999	–	0	99999	0			
12	1	8	9	3	3	0	
7	-1	1	3	7	1	1087208	1097508
17	1	16	0.5625	29	0	3	
99999	–	–	–	0	–	3	7
99999	–	30	0	0			
12	1	9	1	4	4	0	
4	-1	1	4	6	0	1011209	1000509
3	1	16	0.0625	29	43	6	
0	–	–	–	0	–	6	4
99999	–	0	99999	0			
12	1	10	6	6	6	0	
4	-1	1	6	4	1	1094410	1071310
18	1	16	0.375	29	0	6	
99999	–	–	–	0	–	6	4
99999	–	40	0	0			
12	1	11	7	5	5	0	
5	-1	1	5	5	0	1047311	1072111
9	1	16	0.4375	29	14	5	
0	–	–	–	0	–	5	5
99999	–	0	99999	0			
12	1	12	2	4	4	0	
4	-1	1	4	6	0	1020112	1025412
4	1	16	0.125	29	14	6	
0	–	–	–	0	–	6	4
99999	–	0	99999	0			
12	1	13	5	0	0	0	
2	-1	0	0	0	1	1052613	1034713
11	1	16	0.3125	29	0	8	
99999	–	–	–	0	–	0	0
99999	–	14	0	0			
12	1	14	4	7	7	0	
3	-1	1	7	3	1	1053014	1018114
12	1	16	0.25	29	0	7	
99999	–	–	–	0	–	7	3
99999	–	25	0	0			
12	1	15	7	5	5	0	
5	-1	1	5	5	1	1056315	1084215
13	1	16	0.4375	29	0	5	
99999	–	–	–	0	–	5	5
99999	–	34	0	0			
12	1	16	3	0	0	0	
2	-1	0	0	0	1	1070116	1017516

15	1	16	0.1875	29	0	8	
99999	–	–	–	0	–	0	0
99999	–	26	0	0			
12	1	17	8	5	5	0	
5	-1	1	5	5	1	1067317	1091317
14	1	16	0.5	29	0	5	
99999	–	–	–	0	–	5	5
99999	–	25	0	0			
12	1	18	4	3	3	0	
3	-1	1	3	7	0	1047218	1033218
8	1	16	0.25	29	32	7	
0	–	–	–	0	–	7	3
99999	–	0	99999	0			
12	2	1	8	0	6	1	
3	-1	0	0	0	1	1048501	1091801
10	1	16	0.5	–	–	7	–
99999	0	99999	0	99999	0	0	99999
99999	–	–	0				
12	2	2	5	5	12	1	
5	-1	1	5	6	0	1020302	1039502
5	1	16	0.3125	–	–	5	–
99999	99999	0	1	0	5	5	99999
99999	–	–	0				
12	2	3	3	8	14	1	
4	-1	1	8	4	1	1082103	1021003
16	1	16	0.1875	–	–	6	–
99999	0	99999	2	99999	6	4	99999
99999	–	–	0				
12	2	4	6	4	9	1	
4	-1	1	4	8	0	1005204	1065604
1	1	16	0.375	–	–	6	–
99999	99999	0	2	0	6	4	99999
99999	–	–	0				
12	2	5	8	0	0	1	
3	-1	0	0	0	0	1045005	1081205
7	1	16	0.5	–	–	7	–
99999	99999	0	0	0	0	0	99999
99999	–	–	0				
12	2	6	7	0	0	1	
6	-1	0	0	5	0	1010506	1080206
2	1	16	0.4375	–	–	4	–
99999	99999	0	5	0	0	0	99999
99999	–	–	0				
12	2	7	2	0	4	1	
3	-1	0	0	0	0	1039607	1015407
6	1	16	0.125	–	–	7	–
99999	99999	0	0	0	0	0	99999
99999	–	–	0				
12	2	8	2	0	3	1	
3	-1	0	0	0	1	1087208	1010108
17	1	16	0.125	–	–	7	–
99999	0	99999	0	99999	0	0	99999
99999	–	–	0				
12	2	9	1	3	7	1	

3	-1	1	3	12	0	1011209	1013509
3	1	16	0.0625	-	-	7	-
99999	99999	0	5	0	7	3	99999
99999	-	-	0				
12	2	10	4	10	16	1	
5	-1	1	10	5	1	1094410	1022110
18	1	16	0.25	-	-	5	-
99999	0	99999	5	99999	5	5	99999
99999	-	-	0				
12	2	11	4	5	10	1	
5	-1	1	5	10	0	1047311	1039111
9	1	16	0.25	-	-	5	-
99999	99999	0	5	0	5	5	99999
99999	-	-	0				
12	2	12	9	7	11	1	
7	-1	1	7	8	0	1020112	1081912
4	1	16	0.5625	-	-	3	-
99999	99999	0	5	0	3	7	99999
99999	-	-	0				
12	2	13	6	8	8	1	
4	-1	1	8	4	1	1052613	1041313
11	1	16	0.375	-	-	6	-
99999	0	99999	2	99999	6	4	99999
99999	-	-	0				
12	2	14	1	12	19	1	
3	-1	1	12	3	1	1053014	1008914
12	1	16	0.0625	-	-	7	-
99999	0	99999	5	99999	7	3	99999
99999	-	-	0				
12	2	15	7	5	10	1	
6	-1	0	5	0	1	1056315	1073215
13	1	16	0.4375	-	-	4	-
99999	0	99999	5	99999	0	0	99999
99999	-	-	0				
12	2	16	5	6	6	1	
5	-1	1	6	5	1	1070116	1025616
15	1	16	0.3125	-	-	5	-
99999	0	99999	1	99999	5	5	99999
99999	-	-	0				
12	2	17	9	8	13	1	
7	-1	1	8	7	1	1067317	1098517
14	1	16	0.5625	-	-	3	-
99999	0	99999	5	99999	3	7	99999
99999	-	-	0				
12	2	18	3	4	7	1	
4	-1	1	4	8	0	1047218	1030018
8	1	16	0.1875	-	-	6	-
99999	99999	0	2	0	6	4	99999
99999	-	-	0				
13	1	1	10	0	0	0	
2	-1	0	0	0	0	1048001	1086401
10	1	18	0.555555556		29	41	8
0	0	0	0	0	0	0	99999
0	0	99999		1			

13	1	2	5	6	6	0	
4	-1	1	6	4	1	1048302	1032802
11	1	18	0.277777778		29	0	6
99999	-	-	-	0	6	4	
99999	-	11	0		1		
13	1	3	10	0	0	0	
2	-1	0	0	0	1	1066703	1088603
14	1	18	0.555555556		22	0	8
99999	-	-	-	0	0	0	
99999	-	14	0		1		
13	1	4	2	7	7	0	
3	-1	1	7	3	1	1077504	1014604
16	1	18	0.111111111		28	0	7
99999	-	-	-	0	7	3	
99999	-	21	0		1		
13	1	5	8	6	6	0	
4	-1	1	6	4	1	1092005	1081305
20	1	18	0.444444444		29	0	6
99999	-	-	-	0	6	4	
99999	-	34	0		1		
13	1	6	3	7	7	0	
3	-1	1	7	3	1	1058706	1017206
12	1	18	0.166666667		29	0	7
99999	-	-	-	0	7	3	
99999	-	39	0		1		
13	1	7	7	5	5	0	
5	-1	1	5	5	0	1037607	1062307
8	1	18	0.388888889		29	39	5
0	-	-	-	0	5	5	
99999	-	0	99999		1		
13	1	8	7	5	5	0	
5	-1	1	5	5	1	1081208	1062308
17	1	18	0.388888889		29	0	5
99999	-	-	-	0	5	5	
99999	-	42	0		1		
13	1	9	9	7	7	0	
7	-1	1	7	3	0	1031909	1079709
7	1	18	0.5	29	26	3	
0	-	-	-	0	3	7	
99999	-	0	99999		1		
13	1	10	1	4	4	0	
4	-1	1	4	6	0	1009910	1042910
3	1	18	0.055555556		24	37	6
0	-	-	-	0	6	4	
99999	-	0	99999		1		
13	1	11	9	3	3	0	
7	-1	1	3	7	1	1085411	1084511
19	1	18	0.5	28	0	3	
99999	-	-	-	0	3	7	
99999	-	23	0		1		
13	1	12	6	6	6	0	
4	-1	1	6	4	1	1066612	1050112
13	1	18	0.333333333		29	0	6
99999	-	-	-	0	6	4	

99999	-	28	0		1		
13	1	13	3	3	3	0	
3	-1	1	3	7	0	1001813	1052013
1	1	18	0.166666667		29	42	7
0	-	-	-	0	7	3	
99999	-	0	99999		1		
13	1	14	8	4	4	0	
4	-1	1	4	6	0	1028414	1069814
5	1	18	0.444444444		29	45	6
0	-	-	-	0	6	4	
99999	-	0	99999		1		
13	1	15	5	4	4	0	
4	-1	1	4	6	0	1047815	1057415
9	1	18	0.277777778		29	45	6
0	-	-	-	0	6	4	
99999	-	0	99999		1		
13	1	16	4	0	0	0	
4	-1	0	0	0	1	1074616	1032016
15	1	18	0.222222222		29	0	6
99999	-	-	-	0	0	0	
99999	-	7	0		1		
13	1	17	1	6	6	0	
4	-1	1	6	4	1	1084417	1008917
18	1	18	0.055555556		29	0	6
99999	-	-	-	0	6	4	
99999	-	27	0		1		
13	1	18	2	3	3	0	
3	-1	1	3	7	0	1029718	1048918
6	1	18	0.111111111		28	40	7
0	-	-	-	0	7	3	
99999	-	0	99999		1		
13	1	19	6	4	4	0	
4	-1	1	4	6	0	1001819	1061619
2	1	18	0.333333333		29	7	6
0	-	-	-	0	6	4	
99999	-	0	99999		1		
13	1	20	4	0	0	0	
4	-1	0	0	0	0	1010520	1053020
4	1	18	0.222222222		29	42	6
0	-	-	-	0	0	0	
99999	-	0	99999		1		
13	2	1	4	5	5	1	5
0	1	5	5	0	1048001	1023301	10
1	18	0.222222222		-	-	5	-
99999	0	0	0	5	5	99999	
99999	-	-	40	1			
13	2	2	9	7	13	1	7
1	1	7	7	1	1048302	1040102	11
1	18	0.5	-	-	3	-	99999
42	99999	4	3	7	99999		
99999	-	-	0	1			
13	2	3	1	10	10	1	5
0	1	10	5	1	1066703	1003603	14
1	18	0.055555556		-	-	5	-

33	2	99999	5	5	5	99999	
0	-	-	0	1			
13	2	4	10	11	18	1	4
0	1	11	4	1	1077504	1052504	16
1	18	0.555555556		-	-	6	-
99999	40	99999	5	6	4	99999	
99999	-	-	0	1			
13	2	5	7	9	15	1	4
0	1	9	4	1	1092005	1033905	20
1	18	0.388888889		-	-	6	-
99999	42	99999	3	6	4	99999	
99999	-	-	0	1			
13	2	6	3	12	19	1	3
0	1	12	3	1	1058706	1017306	12
1	18	0.166666667		-	-	7	-
99999	40	99999	5	7	3	99999	
99999	-	-	0	1			
13	2	7	2	4	9	1	4
0	1	4	10	0	1037607	1012307	8
1	18	0.111111111		-	-	6	-
99999	0	0	4	6	4	99999	
99999	-	-	40	1			
13	2	8	8	10	15	1	5
0	1	10	5	1	1081208	1036708	17
1	18	0.444444444		-	-	5	-
99999	41	99999	5	5	5	99999	
99999	-	-	0	1			
13	2	9	5	5	12	1	5
0	1	5	10	0	1031909	1042309	7
1	18	0.277777778		-	-	5	-
99999	0	0	5	5	5	99999	
99999	-	-	42	1			
13	2	10	1	5	9	1	5
0	1	5	10	0	1009910	1007410	3
1	18	0.055555556		-	-	5	-
99999	0	0	5	5	5	99999	
99999	-	-	39	1			
13	2	11	4	5	8	1	5
0	1	5	5	1	1085411	1018411	19
1	18	0.222222222		-	-	5	-
99999	37	99999	0	5	5	99999	
99999	-	-	0	1			
13	2	12	6	7	13	1	3
1	1	7	3	1	1066612	1029412	13
1	18	0.333333333		-	-	7	-
20	36	99999	0	7	3	99999	
99999	-	-	0	1			
13	2	13	10	4	7	1	4
0	1	4	11	0	1001813	1085313	1
1	18	0.555555556		-	-	6	-
99999	0	0	5	6	4	99999	
99999	-	-	39	1			
13	2	14	6	3	7	1	3
1	1	3	7	0	1028414	1064114	5

1	18	0.333333333		-	-	7	-
99999	0	0	0	7	3	99999	
99999	-	-	2	1			
13	2	15	7	4	8	1	4
0	1	4	9	0	1047815	1073215	9
1	18	0.388888889		-	-	6	-
99999	0	0	3	6	4	99999	
99999	-	-	42	1			
13	2	16	5	10	10	1	5
0	1	10	5	1	1074616	1028316	15
1	18	0.277777778		-	-	5	-
99999	40	99999	5	5	5	99999	
99999	-	-	0	1			
13	2	17	2	10	16	1	4
0	1	10	4	1	1084417	1004017	18
1	18	0.111111111		-	-	6	-
99999	41	99999	4	6	4	99999	
99999	-	-	0	1			
13	2	18	3	3	6	1	3
0	1	3	12	0	1029718	1016218	6
1	18	0.166666667		-	-	7	-
99999	0	0	5	7	3	99999	
99999	-	-	37	1			
13	2	19	9	7	11	1	7
1	1	7	7	0	1001819	1076319	2
1	18	0.5	-	-	3	-	99999
0	0	4	3	7	99999		
99999	-	-	34	1			
13	2	20	8	5	5	1	5
0	1	5	10	0	1010520	1075920	4
1	18	0.444444444		-	-	5	-
99999	0	0	5	5	5	99999	
99999	-	-	43	1			
14	1	1	2	3	3	0	
3	-1	1	3	7	0	1005201	1043301
1	1	20	0.1	29	29	7	0
0	0	0	0	7	3	99999	0
0	99999		1				
14	1	2	3	4	4	0	
4	-1	1	4	6	0	1035302	1052402
6	1	20	0.15	29	33	6	
0	-	-	-	0	6	4	
99999	-	0	99999		1		
14	1	3	6	7	7	0	
3	-1	1	7	3	1	1058703	1071403
12	1	20	0.3	29	0	7	
99999	-	-	-	0	7	3	
99999	-	29	0		1		
14	1	4	5	5	5	0	
5	-1	1	5	5	0	1044504	1072004
7	1	20	0.25	15	43	5	
0	-	-	-	0	5	5	
99999	-	0	99999		1		
14	1	5	5	5	5	0	

5	-1	1	5	5	1	1052205	1064605
10	1	20	0.25	29	0	5	
99999	-	-	-	0	5	5	
99999	-	36	0		1		
14	1	6	2	7	7	0	
3	-1	1	7	3	1	1056606	1032906
11	1	20	0.1	29	0	7	
99999	-	-	-	0	7	3	
99999	-	29	0		1		
14	1	7	4	5	5	0	
5	-1	1	5	5	0	1047307	1061907
8	1	20	0.2	29	99999	5	
0	-	-	-	0	5	5	
99999	-	0	99999		1		
14	1	8	4	5	5	0	
5	-1	1	5	5	1	1047808	1038708
9	1	20	0.2	29	0	5	
99999	-	-	-	0	5	5	
99999	-	32	0		1		
14	1	9	1	4	4	0	
6	-1	1	4	6	1	1076609	1026609
15	1	20	0.05	29	0	4	
99999	-	-	-	0	4	6	
99999	-	29	0		1		
14	1	10	7	8	8	0	
2	-1	1	8	2	1	1070710	1072910
14	1	20	0.35	28	0	8	
99999	-	-	-	0	8	2	
99999	-	30	0		1		
14	1	11	3	6	6	0	
4	-1	1	6	4	1	1064411	1034011
13	1	20	0.15	29	0	6	
99999	-	-	-	0	6	4	
99999	-	29	0		1		
14	1	12	7	2	2	0	
2	-1	1	2	8	0	1025812	1081912
4	1	20	0.35	28	42	8	
0	-	-	-	0	8	2	
99999	-	0	99999		1		
14	1	13	8	4	4	0	
4	-1	1	4	6	0	1014613	1089413
2	1	20	0.4	29	44	6	
0	-	-	-	0	6	4	
99999	-	0	99999		1		
14	1	14	8	6	6	0	
4	-1	1	6	4	1	1099114	1097214
16	1	20	0.4	29	0	6	
99999	-	-	-	0	6	4	
99999	-	38	0		1		
14	1	15	1	6	6	0	
6	-1	1	6	4	0	1019515	1039315
3	1	20	0.05	28	39	4	
0	-	-	-	0	4	6	
99999	-	0	99999		1		

14	1	16	6	3	3	0	
3	-1	1	3	7	0	1034516	1081516
5	1	20	0.3	29	29	7	
0	–	–	–	0	7	3	
99999	–	0	99999		1		
14	1	17	10	6	6	0	
4	-1	1	6	4	1	2092717	2056617
21	2	20	0.5	29	0	6	
99999	–	–	–	0	6	4	
99999	–	29	0		1		
14	1	18	9	5	5	0	
5	-1	1	5	5	0	2003218	2014618
17	2	20	0.45	29	41	5	
0	–	–	–	0	5	5	
99999	–	0	99999		1		
14	1	19	9	5	5	0	
5	-1	1	5	5	1	2097119	2037719
22	2	20	0.45	29	0	5	
99999	–	–	–	0	5	5	
99999	–	29	0		1		
14	1	20	10	4	4	0	
4	-1	1	4	6	0	2042620	2040120
19	2	20	0.5	29	99999	6	
0	–	–	–	0	6	4	
99999	–	0	99999		1		
14	1	21	11	6	6	0	
6	-1	1	6	4	0	2011121	2093921
18	2	20	0.55	29	40	4	
0	–	–	–	0	4	6	
99999	–	0	99999		1		
14	1	22	11	4	4	0	
6	-1	1	4	6	1	2076522	2059822
20	2	20	0.55	29	0	4	
99999	–	–	–	0	4	6	
99999	–	29	0		1		
14	2	1	7	0	3	1	2
1	0	0	3	0	1005201	1058701	1
1	20	0.35	–	–	8	–	99999
0	0	3	0	0	99999		
99999	–	–	99999	1			
14	2	2	1	0	4	1	2
1	0	0	0	0	1035302	1011602	6
1	20	0.05	–	–	8	–	99999
0	0	0	0	0	99999		
99999	–	–	99999	1			
14	2	3	1	0	7	1	2
1	0	0	0	1	1058703	1001503	12
1	20	0.05	–	–	8	–	99999
99999	99999	0	0	0	99999		
99999	–	–	0	1			
14	2	4	5	2	7	1	2
1	1	2	8	0	1044504	1034104	7
1	20	0.25	–	–	8	–	99999
0	0	0	8	2	99999		

99999	–	–	99999	1			
14	2	5	2	10	15	1	5
0	1	10	5	1	1052205	1002105	10
1	20	0.1	–	–	5	–	99999
99999	99999	5	5	5	99999		
99999	–	–	0	1			
14	2	6	7	3	10	1	2
1	0	3	0	1	1056606	1084306	11
1	20	0.35	–	–	8	–	99999
99999	99999	3	0	0	99999		
99999	–	–	0	1			
14	2	7	6	3	8	1	3
1	1	3	12	0	1047307	1043507	8
1	20	0.3	–	–	7	–	99999
0	0	5	7	3	99999		
99999	–	–	99999	1			
14	2	8	3	9	14	1	6
1	1	9	6	1	1047808	1009908	9
1	20	0.15	–	–	4	–	99999
99999	99999	5	4	6	99999		
99999	–	–	0	1			
14	2	9	4	6	10	1	4
1	1	6	4	1	1076609	1049909	15
1	20	0.2	–	–	6	–	99999
99999	99999	0	6	4	99999		
99999	–	–	0	1			
14	2	10	8	1	9	1	1
1	0	1	0	1	1070710	1099810	14
1	20	0.4	–	–	9	–	99999
99999	99999	1	0	0	99999		
99999	–	–	0	1			
14	2	11	6	12	18	1	3
1	1	12	3	1	1064411	1069011	13
1	20	0.3	–	–	7	–	99999
99999	99999	5	7	3	99999		
99999	–	–	0	1			
14	2	12	2	5	7	1	5
0	1	5	10	0	1025812	1023812	4
1	20	0.1	–	–	5	–	99999
0	0	5	5	5	99999		
99999	–	–	99999	1			
14	2	13	4	4	8	1	4
1	1	4	6	0	1014613	1032313	2
1	20	0.2	–	–	6	–	99999
0	0	0	6	4	99999		
99999	–	–	99999	1			
14	2	14	5	8	14	1	2
1	1	8	2	1	1099114	1054014	16
1	20	0.25	–	–	8	–	99999
99999	99999	0	8	2	99999		
99999	–	–	0	1			
14	2	15	3	6	12	1	6
1	1	6	9	0	1019515	1031215	3
1	20	0.15	–	–	4	–	99999

0	0	5	4	6	99999		
99999	–	–	99999	1			
14	2	16	8	0	3	1	1
1	0	0	1	0	1034516	1090916	5
1	20	0.4	–	–	9	–	99999
0	0	1	0	0	99999		
99999	–	–	99999	1			
14	2	17	9	13	19	1	2
1	1	13	2	1	2092717	2010217	21
2	20	0.45	–	–	8	–	99999
99999	99999	5	8	2	99999		
99999	–	–	0	1			
14	2	18	11	5	10	1	5
1	1	5	5	0	2003218	2064618	17
2	20	0.55	–	–	5	–	99999
0	0	0	5	5	99999		
99999	–	–	99999	1			
14	2	19	10	10	15	1	5
0	1	10	5	1	2097119	2044419	22
2	20	0.5	–	–	5	–	99999
99999	99999	5	5	5	99999		
99999	–	–	0	1			
14	2	20	10	5	9	1	5
0	1	5	10	0	2042620	2056020	19
2	20	0.5	–	–	5	–	99999
0	0	5	5	5	99999		
99999	–	–	99999	1			
14	2	21	9	2	8	1	2
1	1	2	13	0	2011121	2012921	18
2	20	0.45	–	–	8	–	99999
0	0	5	8	2	99999		
99999	–	–	99999	1			
14	2	22	11	5	9	1	5
1	1	5	5	1	2076522	2099722	20
2	20	0.55	–	–	5	–	99999
99999	99999	0	5	5	99999		
99999	–	–	0	1			
15	1	1	9	7	7	0	
7	-1	1	7	3	0	1018701	1079301
2	1	20	0.45	29	44	3	0
0	0	0	0	0	3	7	99999
0	0	99999	2				
15	1	2	5	5	5	0	
5	-1	1	5	5	1	1077102	1058602
14	1	20	0.25	29	0	5	
99999	–	–	–	0	–	5	5
99999	–	99999	0	2			
15	1	3	8	5	5	0	
5	-1	1	5	5	0	1052903	1074703
10	1	20	0.4	29	44	5	
0	–	–	–	0	–	5	5
99999	–	0	99999	2			
15	1	4	11	8	8	0	
2	-1	1	8	2	1	1063404	1098104

12	1	20	0.55	29	0	8	
99999	–	–	–	0	–	8	2
99999	–	43	0	2			
15	1	5	1	3	3	0	
7	-1	1	3	7	1	1079005	1008905
15	1	20	0.05	29	0	3	
99999	–	–	–	0	–	3	7
99999	–	43	0	2			
15	1	6	2	5	5	0	
5	-1	1	5	5	1	1065006	1009106
13	1	20	0.1	29	0	5	
99999	–	–	–	0	–	5	5
99999	–	43	0	2			
15	1	7	3	5	5	0	
5	-1	1	5	5	1	1081907	1024507
16	1	20	0.15	29	0	5	
99999	–	–	–	0	–	5	5
99999	–	43	0	2			
15	1	8	4	9	9	0	
9	-1	1	9	1	0	1036608	1038408
7	1	20	0.2	29	43	1	
0	–	–	–	0	–	1	9
99999	–	0	99999	2			
15	1	9	4	1	1	0	
9	-1	1	1	9	1	1091909	1053909
18	1	20	0.2	29	0	1	
99999	–	–	–	0	–	1	9
99999	–	43	0	2			
15	1	10	8	5	5	0	
5	-1	1	5	5	1	1095310	1087810
21	1	20	0.4	29	0	5	
99999	–	–	–	0	–	5	5
99999	–	43	0	2			
15	1	11	7	6	6	0	
4	-1	1	6	4	1	1082511	1076111
17	1	20	0.35	29	0	6	
99999	–	–	–	0	–	6	4
99999	–	43	0	2			
15	1	12	7	4	4	0	
4	-1	1	4	6	0	1050812	1061412
9	1	20	0.35	29	43	6	
0	–	–	–	0	–	6	4
99999	–	0	99999	2			
15	1	13	2	5	5	0	
5	-1	1	5	5	0	1032913	1028813
4	1	20	0.1	29	43	5	
0	–	–	–	0	–	5	5
99999	–	0	99999	2			
15	1	14	1	7	7	0	
7	-1	1	7	3	0	1060214	1014614
11	1	20	0.05	29	43	3	
0	–	–	–	0	–	3	7
99999	–	0	99999	2			
15	1	15	5	5	5	0	

5	-1	1	5	5	0	1034515	1046015
5	1	20	0.25	26	99999	5	
0	-	-	-	0	-	5	5
99999	-	0	99999	2			
15	1	16	10	6	6	0	
4	-1	1	6	4	1	1092616	1096616
19	1	20	0.5	29	0	6	
99999	-	-	-	0	-	6	4
99999	-	43	0	2			
15	1	17	11	2	2	0	
2	-1	1	2	8	0	1022217	1099917
3	1	20	0.55	29	43	8	
0	-	-	-	0	-	8	2
99999	-	0	99999	2			
15	1	18	3	5	5	0	
5	-1	1	5	5	0	1036518	1029518
6	1	20	0.15	29	43	5	
0	-	-	-	0	-	5	5
99999	-	0	99999	2			
15	1	19	10	4	4	0	
4	-1	1	4	6	0	1038019	1088619
8	1	20	0.5	26	99999	6	
0	-	-	-	0	-	6	4
99999	-	0	99999	2			
15	1	20	6	6	6	0	
4	-1	1	6	4	1	1093720	1070120
20	1	20	0.3	26	0	6	
99999	-	-	-	0	-	6	4
99999	-	99999	0	2			
15	1	21	6	4	4	0	
4	-1	1	4	6	0	1012221	1048221
1	1	20	0.3	26	99999	6	
0	-	-	-	0	-	6	4
99999	-	0	99999	2			
15	1	22	9	3	3	0	
7	-1	1	3	7	1	1099222	1089222
22	1	20	0.45	29	0	3	
99999	-	-	-	0	-	3	7
99999	-	43	0	2			
15	2	1	8	5	12	1	5
0	1	5	10	0	1018701	1066201	2
1	20	0.4	-	-	5	-	99999
99999	0	5	0	5	5	99999	
99999	-	-	2				
15	2	2	9	10	15	1	5
1	1	10	5	1	1077102	1068502	14
1	20	0.45	-	-	5	-	0
0	99999	5	99999	5	5	99999	
99999	-	-	2				
15	2	3	6	0	5	1	3
1	0	0	0	0	1052903	1042303	10
1	20	0.3	-	-	7	-	99999
99999	0	0	0	0	0	99999	
99999	-	-	2				

15	2	4	7	9	17	1	2
1	1	9	2	1	1063404	1061004	12
1	20	0.35	-	-	8	-	0
0	99999	1	99999	8	2	99999	
99999	-	-	2				
15	2	5	6	0	3	1	3
1	0	0	0	1	1079005	1056505	15
1	20	0.3	-	-	7	-	0
0	99999	0	99999	0	0	99999	
99999	-	-	2				
15	2	6	8	10	15	1	5
0	1	10	5	1	1065006	1066406	13
1	20	0.4	-	-	5	-	0
0	99999	5	99999	5	5	99999	
99999	-	-	2				
15	2	7	1	10	15	1	5
0	1	10	5	1	1081907	1005507	16
1	20	0.05	-	-	5	-	0
0	99999	5	99999	5	5	99999	
99999	-	-	2				
15	2	8	7	2	11	1	2
1	1	2	9	0	1036608	1047408	7
1	20	0.35	-	-	8	-	99999
99999	0	1	0	8	2	99999	
99999	-	-	2				
15	2	9	3	5	6	1	0
1	0	5	0	1	1091909	1027509	18
1	20	0.15	-	-	10	-	0
0	99999	5	99999	0	0	99999	
99999	-	-	2				
15	2	10	5	9	14	1	6
0	1	9	6	1	1095310	1041210	21
1	20	0.25	-	-	4	-	0
0	99999	5	99999	4	6	99999	
99999	-	-	2				
15	2	11	4	6	12	1	6
1	1	6	6	1	1082511	1040311	17
1	20	0.2	-	-	4	-	0
0	99999	2	99999	4	6	99999	
99999	-	-	2				
15	2	12	9	5	9	1	5
1	1	5	10	0	1050812	1072612	9
1	20	0.45	-	-	5	-	99999
99999	0	5	0	5	5	99999	
99999	-	-	2				
15	2	13	3	0	5	1	0
1	0	0	5	0	1032913	1015813	4
1	20	0.15	-	-	10	-	99999
99999	0	5	0	0	0	99999	
99999	-	-	2				
15	2	14	11	4	11	1	4
1	1	4	7	0	1060214	1091314	11
1	20	0.55	-	-	6	-	99999
99999	0	1	0	6	4	99999	

99999	–	–	2				
15	2	15	4	6	11	1	6
1	1	6	6	0	1034515	1022715	5
1	20	0.2	–	–	4	–	99999
99999	0	2	0	4	6	99999	
99999	–	–	2				
15	2	16	10	12	18	1	3
0	1	12	3	1	1092616	1070416	19
1	20	0.5	–	–	7	–	0
0	99999	5	99999	7	3	99999	
99999	–	–	2				
15	2	17	10	3	5	1	3
0	1	3	12	0	1022217	1087817	3
1	20	0.5	–	–	7	–	99999
99999	0	5	0	7	3	99999	
99999	–	–	2				
15	2	18	5	6	11	1	6
0	1	6	9	0	1036518	1032018	6
1	20	0.25	–	–	4	–	99999
99999	0	5	0	4	6	99999	
99999	–	–	2				
15	2	19	2	6	10	1	6
1	1	6	9	0	1038019	1014119	8
1	20	0.1	–	–	4	–	99999
99999	0	5	0	4	6	99999	
99999	–	–	2				
15	2	20	2	9	15	1	6
1	1	9	6	1	1093720	1014020	20
1	20	0.1	–	–	4	–	0
0	99999	5	99999	4	6	99999	
99999	–	–	2				
15	2	21	1	5	9	1	5
0	1	5	10	0	1012221	1006021	1
1	20	0.05	–	–	5	–	99999
99999	0	5	0	5	5	99999	
99999	–	–	2				
15	2	22	11	7	10	1	4
1	1	7	4	1	1099222	1086422	22
1	20	0.55	–	–	6	–	0
0	99999	1	99999	6	4	99999	
99999	–	–	2				
16	1	1	5	5	5	0	
5	-1	1	5	5	0	1009201	1043001
3	1	20	0.25	29	99999	5	0
0	0	0	0	0	5	5	99999
0	0	99999	2				
16	1	2	6	0	0	0	
3	-1	0	0	0	0	1026402	1043302
5	1	20	0.3	29	99999	7	
0	–	–	–	0	–	0	0
99999	–	0	99999	2			
16	1	3	11	5	5	0	
5	-1	1	5	5	1	1070403	1098103
18	1	20	0.55	29	0	5	

99999	–	–	–	0	–	5	5
99999	–	99999	0	2			
16	1	4	5	5	5	0	
5	-1	1	5	5	1	1063004	1058704
15	1	20	0.25	29	0	5	
99999	–	–	–	0	–	5	5
99999	–	99999	0	2			
16	1	5	8	5	5	0	
5	-1	1	5	5	1	1084105	1084505
20	1	20	0.4	29	0	5	
99999	–	–	–	0	–	5	5
99999	–	99999	0	2			
16	1	6	1	5	5	0	
5	-1	1	5	5	0	1011306	1010206
4	1	20	0.05	29	99999	5	
0	–	–	–	0	–	5	5
99999	–	0	99999	2			
16	1	7	4	4	4	0	
4	-1	1	4	6	0	1058207	1041707
11	1	20	0.2	29	99999	6	
0	–	–	–	0	–	6	4
99999	–	0	99999	2			
16	1	8	10	8	8	0	
2	-1	1	8	2	1	1069108	1096108
17	1	20	0.5	29	0	8	
99999	–	–	–	0	–	8	2
99999	–	99999	0	2			
16	1	9	7	5	5	0	
5	-1	1	5	5	1	1062209	1071709
14	1	20	0.35	29	0	5	
99999	–	–	–	0	–	5	5
99999	–	99999	0	2			
16	1	10	11	5	5	0	
5	-1	1	5	5	0	1026810	1090710
6	1	20	0.55	29	99999	5	
0	–	–	–	0	–	5	5
99999	–	0	99999	2			
16	1	11	3	5	5	0	
5	-1	1	5	5	1	1088011	1051811
21	1	20	0.15	29	0	5	
99999	–	–	–	0	–	5	5
99999	–	99999	0	2			
16	1	12	9	5	5	0	
5	-1	1	5	5	1	1071612	1093612
19	1	20	0.45	29	0	5	
99999	–	–	–	0	–	5	5
99999	–	99999	0	2			
16	1	13	2	5	5	0	
5	-1	1	5	5	1	1088313	1018113
22	1	20	0.1	29	0	5	
99999	–	–	–	0	–	5	5
99999	–	99999	0	2			
16	1	14	10	2	2	0	
2	-1	1	2	8	0	1027814	1083614

7	1	20	0.5	29	99999	8	
0	–	–	–	0	–	8	2
99999	–	0	99999	2			
16	1	15	6	0	0	0	
3	-1	0	0	0	1	1066215	1066515
16	1	20	0.3	29	0	7	
99999	–	–	–	0	–	0	0
99999	–	99999	0	2			
16	1	16	3	5	5	0	
5	-1	1	5	5	0	1002616	1033516
1	1	20	0.15	29	99999	5	
0	–	–	–	0	–	5	5
99999	–	0	99999	2			
16	1	17	4	6	6	0	
4	-1	1	6	4	1	1058617	1053317
12	1	20	0.2	29	0	6	
99999	–	–	–	0	–	6	4
99999	–	99999	0	2			
16	1	18	9	5	5	0	
5	-1	1	5	5	0	1043118	1080418
9	1	20	0.45	29	99999	5	
0	–	–	–	0	–	5	5
99999	–	0	99999	2			
16	1	19	7	5	5	0	
5	-1	1	5	5	0	1054819	1064919
10	1	20	0.35	29	99999	5	
0	–	–	–	0	–	5	5
99999	–	0	99999	2			
16	1	20	2	5	5	0	
5	-1	1	5	5	0	1009120	1023720
2	1	20	0.1	29	99999	5	
0	–	–	–	0	–	5	5
99999	–	0	99999	2			
16	1	21	8	5	5	0	
5	-1	1	5	5	0	1029821	1066621
8	1	20	0.4	29	99999	5	
0	–	–	–	0	–	5	5
99999	–	0	99999	2			
16	1	22	1	5	5	0	
5	-1	1	5	5	1	1060622	1001422
13	1	20	0.05	29	0	5	
99999	–	–	–	0	–	5	5
99999	–	99999	0	2			
16	2	1	2	7	12	1	7
1	1	7	7	0	1009201	1001901	3
1	20	0.1	–	–	3	–	99999
99999	0	4	0	3	7	99999	
99999	–	–	2				
16	2	2	11	0	0	1	2
1	0	0	1	0	1026402	1086802	5
1	20	0.55	–	–	8	–	99999
99999	0	1	0	0	0	99999	
99999	–	–	2				
16	2	3	9	5	10	1	5

0	1	5	5	1	1070403	1064203	18
1	20	0.45	-	-	5	-	0
0	99999	0	99999	5	5	99999	
99999	-	-	2				
16	2	4	3	10	15	1	5
0	1	10	5	1	1063004	1006204	15
1	20	0.15	-	-	5	-	0
0	99999	5	99999	5	5	99999	
99999	-	-	2				
16	2	5	6	8	13	1	5
0	1	8	5	1	1084105	1024005	20
1	20	0.3	-	-	5	-	0
0	99999	3	99999	5	5	99999	
99999	-	-	2				
16	2	6	10	5	10	1	5
0	1	5	7	0	1011306	1068106	4
1	20	0.5	-	-	5	-	99999
99999	0	2	0	5	5	99999	
99999	-	-	2				
16	2	7	6	5	9	1	5
0	1	5	8	0	1058207	1041907	11
1	20	0.3	-	-	5	-	99999
99999	0	3	0	5	5	99999	
99999	-	-	2				
16	2	8	11	1	9	1	2
1	0	1	0	1	1069108	1083908	17
1	20	0.55	-	-	8	-	0
0	99999	1	99999	0	0	99999	
99999	-	-	2				
16	2	9	10	7	12	1	5
0	1	7	5	1	1062209	1073809	14
1	20	0.5	-	-	5	-	0
0	99999	2	99999	5	5	99999	
99999	-	-	2				
16	2	10	3	5	10	1	5
0	1	5	10	0	1026810	1007310	6
1	20	0.15	-	-	5	-	99999
99999	0	5	0	5	5	99999	
99999	-	-	2				
16	2	11	5	4	9	1	6
0	1	4	6	1	1088011	1019911	21
1	20	0.25	-	-	4	-	0
0	99999	0	99999	4	6	99999	
99999	-	-	2				
16	2	12	8	9	14	1	6
1	1	9	6	1	1071612	1063212	19
1	20	0.4	-	-	4	-	0
0	99999	5	99999	4	6	99999	
99999	-	-	2				
16	2	13	7	10	15	1	5
0	1	10	5	1	1088313	1062813	22
1	20	0.35	-	-	5	-	0
0	99999	5	99999	5	5	99999	
99999	-	-	2				

16	2	14	7	5	7	1	5
0	1	5	10	0	1027814	1043714	7
1	20	0.35	-	-	5	-	99999
99999	0	5	0	5	5	99999	
99999	-	-	2				
16	2	15	1	6	6	1	4
0	1	6	4	1	1066215	1002915	16
1	20	0.05	-	-	6	-	0
0	99999	0	99999	6	4	99999	
99999	-	-	2				
16	2	16	5	6	11	1	6
0	1	6	4	0	1002616	1030316	1
1	20	0.25	-	-	4	-	99999
99999	0	0	0	4	6	99999	
99999	-	-	2				
16	2	17	2	7	13	1	7
1	1	7	7	1	1058617	1005017	12
1	20	0.1	-	-	3	-	0
0	99999	4	99999	3	7	99999	
99999	-	-	2				
16	2	18	1	4	9	1	4
0	1	4	6	0	1043118	1001418	9
1	20	0.05	-	-	6	-	99999
99999	0	0	0	6	4	99999	
99999	-	-	2				
16	2	19	9	5	10	1	5
0	1	5	5	0	1054819	1052819	10
1	20	0.45	-	-	5	-	99999
99999	0	0	0	5	5	99999	
99999	-	-	2				
16	2	20	4	5	10	1	5
0	1	5	10	0	1009120	1010020	2
1	20	0.2	-	-	5	-	99999
99999	0	5	0	5	5	99999	
99999	-	-	2				
16	2	21	8	6	11	1	6
1	1	6	9	0	1029821	1047621	8
1	20	0.4	-	-	4	-	99999
99999	0	5	0	4	6	99999	
99999	-	-	2				
16	2	22	4	10	15	1	5
0	1	10	5	1	1060622	1012422	13
1	20	0.2	-	-	5	-	0
0	99999	5	99999	5	5	99999	
99999	-	-	2				
