# Supplementary material for: Moral licensing, instrumental apology and insincerity aversion: Taking Immanuel Kant to the lab
Source: PLoS One. 2018 Nov 8;13(11):e0206878. doi: 10.1371/journal.pone.0206878 (PMC6224065; doi:10.1371/journal.pone.0206878)
Supplement: S2 File — Protocols: http://dx.doi.org/10.17504/protocols.io.tutenwn. (PDF) [file pone.0206878.s002.pdf]

# Instruction Protocols: Moral Licensing, Instrumental Apology and Insincerity Aversion: Taking Immanuel Kant to the Lab

Elias Khalil[1], Nick Feltovich[1]

[1]Monash University

dx.doi.org/10.17504/protocols.io.tutenwn

Elias Khalil

ABSTRACT

***Moral licensing*** or, what is equivalently called, “self-licensing” is the use of a Good Act instrumentally: to cover up a Bad Act. This paper’s thesis is that moral licensing is inextricably the same as “instrumental apology,” i.e., bad-faith apology. For instance, a decision maker may issue an apology (Good Act) after committing a Bad Act. But if the decision maker uses the apology instrumentally, he or she is using the apology to justify the Bad Act and, hence, the apology is insincere. Sincerity is the fine line between good-faith apology or, more generally, Good Act, on one hand, and instrumental apology or, more generally, moral licensing, on the other. According to Kantian ethics, the sincerity behind an act, and not just the consequence, matters. This pits Kant against the utilitarian view, which downplays intentions, and focuses on consequences. We take Kant to the lab. Participants play a modified ultimatum game, where proposers in some treatments have the option to issue apology messages, and responders have both costly and costless options for rewarding or punishing. We introduce different treatments of the apology message to allow for responders to form doubt about the sincerity of the apology messages. Our results support the Kantian position: responders exhibit “insincerity aversion” by punishing proposers, when such punishment is costless to them, once they become suspicious of the sincerity of the apology of the proposers.

THIS PROTOCOL ACCOMPANIES THE FOLLOWING PUBLICATION

Khalil, Elias L. and Nick Feltovich. "Moral Licensing, Instrumental Apology and Insincerity Aversion: Taking Immanuel Kant to the Lab." PLOS One, under review, 2018.
